# Deletion in Abstract Voronoi Diagrams in Expected Linear Time and Related Problems

**DOI:** 10.1007/s00454-022-00463-z

**Published:** 2023-03-25

**Authors:** Kolja Junginger, Evanthia Papadopoulou

**Affiliations:** Faculty of Informatics, USI Università della Svizzera italiana, Lugano, Switzerland

**Keywords:** Abstract Voronoi diagram, Linear-time algorithm, Randomized incremental construction, Backwards analysis, Site-deletion, Higher-order Voronoi diagram, Farthest Voronoi diagram, 68W05, 68U05

## Abstract

Updating an abstract Voronoi diagram in linear time, after deletion of one site, has been an open problem in a long time; similarly, for any concrete Voronoi diagram of generalized (non-point) sites. In this paper we present a simple, expected linear-time algorithm to update an abstract Voronoi diagram after deletion of one site. To achieve this result, we use the concept of a Voronoi-like diagram, a relaxed Voronoi structure of independent interest. Voronoi-like diagrams serve as intermediate structures, which are considerably simpler to compute, thus, making an expected linear-time construction possible. We formalize the concept and prove that it is robust under insertion, therefore, enabling its use in incremental constructions. The time-complexity analysis introduces a variant to backwards analysis, which is applicable to order-dependent structures. We further extend the technique to compute in expected linear time: the order-$(k\,{+}\,1)$ subdivision within an order-*k* Voronoi region, and the farthest abstract Voronoi diagram, after the order of its regions at infinity is known.

## Introduction

The Voronoi diagram of a set *S* of *n* simple geometric objects, called sites, is a versatile geometric partitioning structure that reveals proximity information among the input sites. Classic variants include the *nearest-neighbor*, the *farthest-site*, and the *order*-*k* Voronoi diagram of the set *S*. Abstract Voronoi diagrams [11] offer a unifying framework to many concrete and fundamental instances. Voronoi diagrams have been well investigated and many optimal construction algorithms exist in various cases. For more information, see, e.g., the book of Aurenhammer et al. [2], and the book of Okabe et al. [17] for a wealth of applications.

For certain Voronoi diagrams with a tree structure, linear-time algorithms have been well known to exist for their construction, see e.g., [1, 7, 8, 13]. The first linear-time technique was introduced by Aggarwal et al. [1] for the Voronoi diagram of points in convex position, given the order of points along their convex hull. The same technique can be used to derive linear-time algorithms for other fundamental problems: (1) updating a Voronoi diagram of points after deletion of one site in time linear to the number of Voronoi neighbors of the deleted site; (2) computing the order-$(k{+}1)$ subdivision within an order-*k* Voronoi region; (3) computing the farthest Voronoi diagram of point-sites in linear time, given their convex hull. A much simpler randomized technique for the same problems was introduced by Chew [7]. The medial axis of a simple polygon is another well-known problem that admits a linear-time construction, as shown by Chin et al. [8].

Surprisingly, no linear-time constructions have been known for any of the problems (1)–(3) for Voronoi diagrams involving non-point sites, and similarly for abstract Voronoi diagrams. Under restrictions, Klein and Lingas [13] adapted the linear-time approach of [1] to the abstract framework showing that a *Hamiltonian abstract Voronoi diagram* can be computed in linear time, given the order of Voronoi regions along an unbounded simple curve, which visits each region *exactly once* and can intersect each bisector only once. This construction has been extended recently to include some forest structures within a given domain [4], under similar restrictions, where no region can have multiple faces and each bisector can intersect this domain in one component.

In this paper we consider the fundamental problem of site-deletion in abstract Voronoi diagrams and provide a simple expected linear-time technique to achieve this task. We work in the framework of abstract Voronoi diagrams so that we can simultaneously address all the concrete instances that fall under their umbrella. After deletion (1), we extend the randomized linear-time technique to the remaining problems: (2) computing the order-$(k\,{+}\,1)$ subdivision within an order-*k* abstract Voronoi region; and (3) computing the farthest abstract Voronoi diagram after the order of its faces at infinity is known. The latter sequence of faces can be computed in time $O(n\log n)$. To the best of our knowledge, no deterministic linear-time technique is yet known for these problems.

To achieve our goal, we define the *Voronoi-like diagram*, a relaxed Voronoi structure, which is interesting in its own right. *Voronoi-like regions* are supersets of real Voronoi regions, and their boundaries correspond to simple *monotone paths* in the arrangement of the underlying bisector system (see Definition 3.1). We prove the correctness and uniqueness of this structure, and use it to derive a simple randomized incremental algorithm to address the above problems in linear expected time.

An earlier attempt towards a linear-time construction for the farthest-segment Voronoi diagram appeared in [10] following a different geometric formulation, which does not extend to the abstract setting. A preliminary version of the present paper, regarding site deletion in abstract Voronoi diagrams, appeared in [9]. In three dimensions, site-deletion in Delaunay triangulations of point-sites, as inspired by the randomized approach of Chew [7], has been considered in [6].

**Fig. 1:**
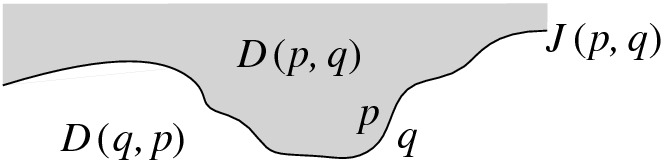
A bisector *J*(*p*, *q*) and its two dominance regions; *D*(*p*, *q*) is shown shaded

*Abstract Voronoi diagrams (AVDs).* These diagrams were introduced by Klein [11]. Instead of sites and distance measures, they are defined in terms of bisecting curves that satisfy some simple combinatorial properties. Given a set *S* of *n* abstract sites, the bisector *J*(*p*, *q*) of two sites $p,q \in S$ is an unbounded Jordan curve, homeomorphic to a line, that divides the plane into two open domains: the *dominance region of* *p*, *D*(*p*, *q*) (having label *p*), and the *dominance region of* *q*, *D*(*q*, *p*) (having label *q*), see Fig. 1. The *Voronoi region* of *p* is

$$\begin{aligned} {{\,\textrm{VR}\,}}(p,S) \,=\! \bigcap _{q \in S \setminus \{p\}}\!D(p,q). \end{aligned}$$

The (*nearest-neighbor*) *Voronoi diagram* of *S* is

$$\begin{aligned} \mathcal {V}(S) = {\mathbb {R}}^2\setminus \bigcup _{p \in S}{{\,\textrm{VR}\,}}(p, S). \end{aligned}$$

Following the traditional model of AVDs (see, e.g., [3, 4, 11]) the bisector system is assumed to satisfy the following axioms, for every subset $S' \subseteq S$:

1. Each Voronoi region ${{\,\textrm{VR}\,}}(p, S')$ is non-empty and path-connected.
2. Each point in the plane belongs to the closure of a Voronoi region ${{\,\textrm{VR}\,}}(p, S')$.
3. Each bisector *J*(*p*, *q*) is an unbounded curve, which after stereographic projection to the sphere can be completed to a closed Jordan curve through the north pole.
4. Any two bisectors *J*(*p*, *q*) and *J*(*r*, *t*) intersect transversally and in a finite number of points. (It is possible to relax this axiom, see [12]).

The abstract Voronoi diagram $\mathcal {V}(S)$ is a plane graph of structural complexity *O*(*n*) whose regions are simply connected. It can be computed in time $O(n\log n)$, randomized [14] or deterministic [11].

To update $\mathcal {V}(S)$ after deleting one site $s\in S$, we need to compute $\mathcal {V}(S\setminus \{s\})$ within ${{\,\textrm{VR}\,}}(s,S)$. This diagram is a tree, if ${{\,\textrm{VR}\,}}(s,S)$ is bounded, and a forest otherwise. However, its regions can be disconnected, i.e., one region may consist of multiple faces. The site-occurrences along $\partial {{\,\textrm{VR}\,}}(s,S)$ form a Davenport–Schinzel sequence of order 2. Disconnected regions introduce severe complications which differentiate the problem from its counterpart on point-sites. For example, let $S'\subset S\setminus \{s\}$; the diagram $\mathcal {V}(S') \cap {{\,\textrm{VR}\,}}(s, S' \cup \{s\})$ may contain faces that do not even appear in $\mathcal {V}(S\setminus \{s\})\cap {{\,\textrm{VR}\,}}(s,S)$, and conversely, an arbitrary sub-sequence of arcs on $\partial {{\,\textrm{VR}\,}}(s,S)$ need not be related to any Voronoi diagram of sites in *S*. At a first sight, a linear-time algorithm may seem infeasible.

*Our results.* In this paper we formalize the concept of a *Voronoi-like diagram*, a relaxed Voronoi structure defined as an acyclic graph (a tree or forest) in the arrangement of the underlying bisector system, and prove that it is well defined and unique. This structure provides a tool to deal with disconnected Voronoi regions, and thus, address the site-deletion problem efficiently. We envision that it will be useful in other cases of Voronoi diagrams with disconnected regions as well.

Given a Voronoi-like diagram, we define an *insertion operation* and prove its correctness. This makes a simple randomized incremental construction possible. The time analysis of the randomized algorithm is non-standard because the intermediate Voronoi-like structures are order-dependent. We give a technique, which offers a simple variant to backwards analysis that can be applied to order-dependent structures. We partition the set of permutations of length *i* into manageable groups of *i* permutations each, and show that the time complexity of step *i* in each group is *O*(*i*). We can then conclude that step *i* is performed in expected *O*(1) time.

In this paper we focus on site-deletion, and compute $\mathcal {V}(S\setminus \{s\})\cap {{\,\textrm{VR}\,}}(s,S)$ in expected time linear in the number of Voronoi neighbors of the deleted site. We also extend the approach to address the aforementioned related problems for the order-*k* and the farthest abstract Voronoi diagram, problems (2) and (3), respectively.

Examples of concrete diagrams that fall under the AVD umbrella, and thus, can benefit from our approach include: disjoint line segments and disjoint convex polygons of constant size in the $L_p$ norms, or under the Hausdorff metric; point-sites in any convex distance metric or the Karlsruhe metric; additively weighted points that have non-enclosing circles; power diagrams with non-enclosing circles.

This paper is organized as follows. Section 2 provides background on abstract Voronoi diagrams. Section 3 formulates the Voronoi-like diagram, which is implied by a subset of $\partial {{\,\textrm{VR}\,}}(s,S)$, given a fixed site $s\in S$. Section 4 defines an insertion operation on a Voronoi-like diagram and proves its correctness. Section 5 proves the uniqueness of the Voronoi-like diagram of a boundary curve. Section 6 outlines the simple randomized incremental construction and proves its time complexity. To this goal, Sect. 6.1 gives a variant of backwards analysis that is applicable to order-dependent structures. To follow the algorithm in Sect. 6 only the basic definitions in Sect. 3 are needed; the correctness and uniqueness proofs of the previous sections are not necessary to follow the algorithm, and thus, they can be skipped. Sections 7 and 8 extend the technique further to the order-*k* and farthest abstract Voronoi diagram respectively. Section 9 gives concluding remarks.

## Preliminaries

Let *S* be a set of *n* abstract *sites* (a set of indices) that define an *admissible* system of bisectors in the plane $\mathcal {J}= \{J(p,q):p\ne q \in S\}$. $\mathcal {J}$ fulfills axioms (A1)–(A4), as given in Sect. 1, for every $S'\subseteq S$.

**Fig. 2:**
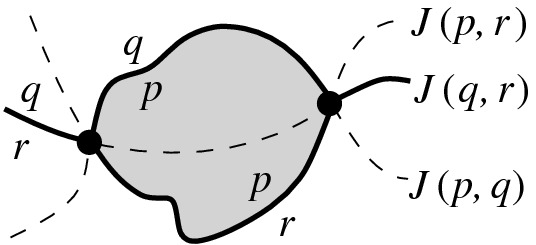
The Voronoi diagram of three sites, if related bisectors (dashed lines) itersect twice; ${{\,\textrm{VR}\,}}(p,\{p,q,r\})$ is shown shaded

Bisectors in $\mathcal {J}$ that have a site *p* in common are called *p*-*related* or simply *related*. Any two related bisectors can intersect at most twice [11, Lem. 3.5.2.5]. When two related bisectors *J*(*p*, *q*) and *J*(*p*, *r*) intersect, bisector *J*(*q*, *r*) also intersects with them at the same point(s), which are the Voronoi vertices of the diagram $\mathcal {V}(\{p,q,r\})$. The Voronoi diagram of three sites $\mathcal {V}(\{p,q,r\})$ may have at most two Voronoi vertices, see Fig. 2. The set of all *p*-related bisectors that involve sites in any $S'\subseteq S$ is denoted $\mathcal {J}_{p,S'} = \{J(p,q):q \in S',\,q \ne p\}$.

Let ${{\,\textrm{VR}\,}}(s,S)$ be the Voronoi region of a site $s\in S$. Although ${{\,\textrm{VR}\,}}(s,S)$ is simply connected, the sites in $S\setminus \{s\}$ appearing along the boundary $\partial {{\,\textrm{VR}\,}}(s,S)$ may repeat, forming a Davenport–Schinzel sequence of order 2. This is because *s*-related bisectors can intersect at most twice, and thus, [21, Thm. 5.7] applies. This is a fundamental difference from the classic case of point-sites in the Euclidean plane, where bisectors are straight-lines, therefore, they intersect at most once, and no site repetition can occur along the boundary of a Voronoi region.

Suppose we delete the site $s\in S$ from $\mathcal {V}(S)$. To update the Voronoi diagram after the deletion of *s*, we need to compute $\mathcal {V}(S\setminus \{s\})$ within the Voronoi region ${{\,\textrm{VR}\,}}(s,S)$, i.e., compute $\mathcal {V}(S\setminus \{s\})\cap {{\,\textrm{VR}\,}}(s,S)$. We first characterize the structure of this diagram in the following lemma. An alternative proof can also be derived from the order-*k* counterpart [5], which appeared after the preliminary version of this paper [9].

### Lemma 2.1

**Fig. 3:**
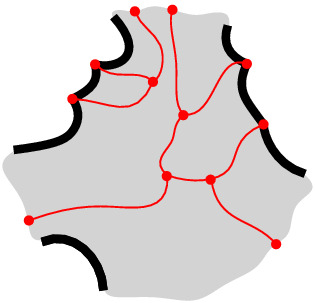
$\mathcal {V}(S \setminus \{s\}) \cap {{\,\textrm{VR}\,}}(s,S)$ in red; $\partial {{\,\textrm{VR}\,}}(s,S)$ is shown in bold black

$\mathcal {V}(S\setminus \{s\})\cap {{\,\textrm{VR}\,}}(s,S)$ is a forest having exactly one face for each Voronoi edge of $\partial {{\,\textrm{VR}\,}}(s,S)$. Its leaves are the Voronoi vertices of $\partial {{\,\textrm{VR}\,}}(s,S)$, and points at infinity, if ${{\,\textrm{VR}\,}}(s,S)$ is unbounded (see Fig. 3). If ${{\,\textrm{VR}\,}}(s,S)$ is bounded then $\mathcal {V}(S\setminus \{s\})\cap {{\,\textrm{VR}\,}}(s,S)$ is a tree.

### Proof

Every face in $\mathcal {V}(S\setminus \{s\})\cap {{\,\textrm{VR}\,}}(s,S)$ must touch the boundary $\partial {{\,\textrm{VR}\,}}(s,S)$ because Voronoi regions are non-empty and connected; this implies that the diagram is a forest. Every Voronoi edge $e \subseteq J(s,p)$ on $\partial {{\,\textrm{VR}\,}}(s,S)$ must be entirely in ${{\,\textrm{VR}\,}}(p,S \setminus \{s\})$. Thus, no leaf can lie in the interior of a Voronoi edge of $\partial {{\,\textrm{VR}\,}}(s,S)$. On the other hand, each Voronoi vertex of $\partial {{\,\textrm{VR}\,}}(s,S)$ must be a leaf of the diagram as its incident edges are induced by different sites.

**Fig. 4:**
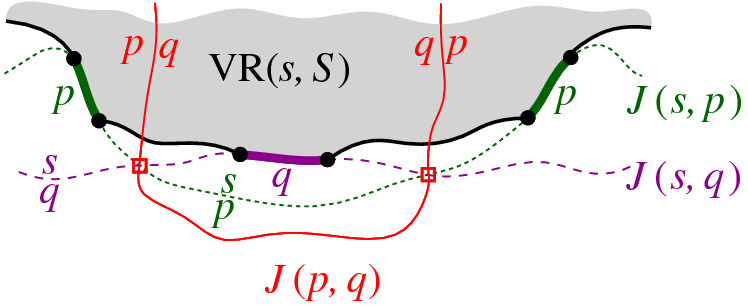
${{\,\textrm{VR}\,}}(p,S \setminus \{s\}) \cap {{\,\textrm{VR}\,}}(s,S)$ cannot be connected because of *J*(*p*, *q*)

Now we show that no two edges of $\partial {{\,\textrm{VR}\,}}(s,S)$ can be incident to the same face of $\mathcal {V}(S \setminus \{s\}) \cap {{\,\textrm{VR}\,}}(s,S)$. Consider two edges on $\partial {{\,\textrm{VR}\,}}(s,S)$ induced by the same site $p\in S \setminus \{s\}$. Then there exists an edge between them, induced by a site $q\ne p$, such that the bisector *J*(*s*, *q*) has exactly two intersections with *J*(*p*, *s*) as shown in Fig. 4. The bisector *J*(*p*, *q*) intersects with them at the same two points. Since the bisector system is admissible, and thus ${{\,\textrm{VR}\,}}(p,\{s,p,q\})$ is connected, *J*(*p*, *q*) connects these endpoints through $D(p,s)\cap D(q,s)$ as shown in Fig. 4, thus, $J(p,q) \cap {{\,\textrm{VR}\,}}(s,\{s,p,q\})$ consists of two unbounded connected components. This implies that $D(p,q)\cap {{\,\textrm{VR}\,}}(s,S)$ must have two disjoint faces, each of which is incident to exactly one of the two edges of *p*. Thus, ${{\,\textrm{VR}\,}}(p,S \setminus \{s\}) \cap {{\,\textrm{VR}\,}}(s,S)$ cannot be connected and the two edges of *p* must be incident to different faces of $\mathcal {V}(S\setminus \{s\})\cap {{\,\textrm{VR}\,}}(s,S)$.

If ${{\,\textrm{VR}\,}}(s,S)$ is unbounded, two consecutive edges of $\partial {{\,\textrm{VR}\,}}(s,S)$ can extend to infinity, in which case there is at least one edge of $\mathcal {V}(S\setminus \{s\})\cap {{\,\textrm{VR}\,}}(s,S)$ extending to infinity between them; thus, leaves can be points at infinity. If ${{\,\textrm{VR}\,}}(s,S)$ is bounded, all leaves of $\mathcal {V}(S\setminus \{s\})\cap {{\,\textrm{VR}\,}}(s,S)$ must lie on $\partial {{\,\textrm{VR}\,}}(s,S)$. Since no face is incident to more than one edge of $\partial {{\,\textrm{VR}\,}}(s,S)$, in this case $\mathcal {V}(S\setminus \{s\})\cap {{\,\textrm{VR}\,}}(s,S)$ cannot be disconnected, and thus is a tree. $\square$

**Fig. 5:**
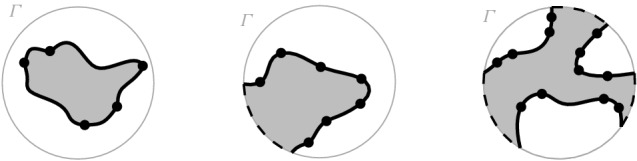
The domain of computation ${{\,\textrm{VR}\,}}(s,S) \cap D_\varGamma$ (shaded)

Let $\varGamma$ be a closed Jordan curve in the plane large enough to enclose all the intersections of bisectors in $\mathcal {J}$, and such that each bisector intersects $\varGamma$ exactly twice and transversally. To avoid dealing with infinity, and without any loss of generality, we restrict all computations within $\varGamma$.^1^ The curve $\varGamma$ can be interpreted as $J(p,s_\infty )$, for any $p \in S$, where $s_\infty$ is an additional site at infinity. Let $D_\varGamma$ denote the portion of the plane enclosed by $\varGamma$. The domain of computation is ${{\,\textrm{VR}\,}}(s,S) \cap D_\varGamma$ and Fig. 5 illustrates possible cases.

We first make some observations regarding an admissible bisector system, which we then use as tools in the proofs throughout this paper.

### Definition 2.2

**Fig. 6:**
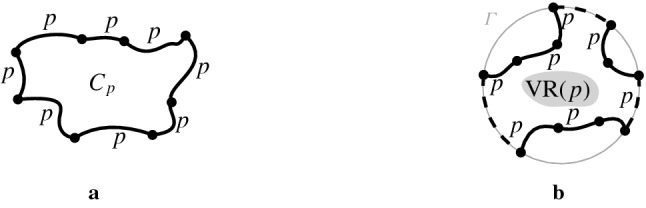
**a** A *p*-inverse cycle. **b** A *p*-cycle

Let $C_p$ be a cycle of *p*-related bisectors in the arrangement of bisectors $\mathcal {J}\cup \{\varGamma \}$, see Fig. 6. If the label *p* appears inside the cycle, for every edge of $C_p$, then $C_p$ is called a *p*-*cycle*. If the label *p* appears on the outside of the cycle for every edge in $C_p$, then $C_p$ is called *p*-*inverse*.

Recall that $\varGamma$ can be considered a *p*-related bisector, for any site $p\in S$, where the label *p* is in the interior of $\varGamma$. Thus, a *p*-cycle may contain arcs of $\varGamma$, while a *p*-inverse cycle cannot contain any $\varGamma$ arcs.

### Lemma 2.3

In an admissible bisector system there is no *p*-inverse cycle.

### Proof

Suppose a *p*-inverse cycle exists in the admissible bisector system. Let $C_p$ denote a minimal such cycle, where no *p*-related bisector may intersect the interior of the cycle, which is denoted by $D_p$. Such a minimal cycle must exist, because if a bisector *J*(*p*, *q*) intersects $D_p$, then it defines another (smaller) *p*-inverse cycle that is contained in $C_p\cup D_p$, whose interior is not intersected by *J*(*p*, *q*). Let $S'\subseteq S$ denote the set of sites that define the edges of $C_p$. Considering $S'$, the farthest Voronoi region of *p* is $\text{ FVR }(p,S') = \bigcap _{q \in S' \setminus \{p\}}D(q,p)$. By its definition, $D_p$ must be identical to one face of $\text{ FVR }(p,S')$. Since farthest Voronoi regions must be unbounded [3, 16], we derive a contradiction. $\square$

The following *transitivity lemma* is a consequence of transitivity of dominance regions [3, Lem. 2] and the fact that bisectors *J*(*p*, *q*), *J*(*q*, *r*), *J*(*p*, *r*) intersect at the same point(s). Let $\overline{X}$ denote the closure of a region *X*.

### Lemma 2.4

Suppose $z \in \mathbb {R}^2$ and $p,q,r \in S$. If $z \in D(p,q)$ and $z \in \overline{D(q,r)}$, then $z \in D(p,r)$.

We make a general position assumption that no three *p*-related bisectors intersect at the same point. This implies that Voronoi vertices have degree 3.

## Problem Formulation, Definitions and Properties

Consider the Voronoi region ${{\,\textrm{VR}\,}}(s,S)$ for a fixed site $s\in S$. Let  denote the sequence of Voronoi edges on the boundary of this region within the domain $D_\varGamma$, i.e., .  is a cyclically ordered set of *arcs*, where each arc is a piece of an *s*-related bisector defining a Voronoi edge on the boundary of ${{\,\textrm{VR}\,}}(s,S)$. The arcs in  are called *core arcs*. Note that a single site in $S\setminus \{s\}$ may induce several of the core arcs in . For any arc , let $s_\alpha$ denote the site in *S* such that $\alpha \subseteq J(s,s_\alpha )$.

**Fig. 7:**
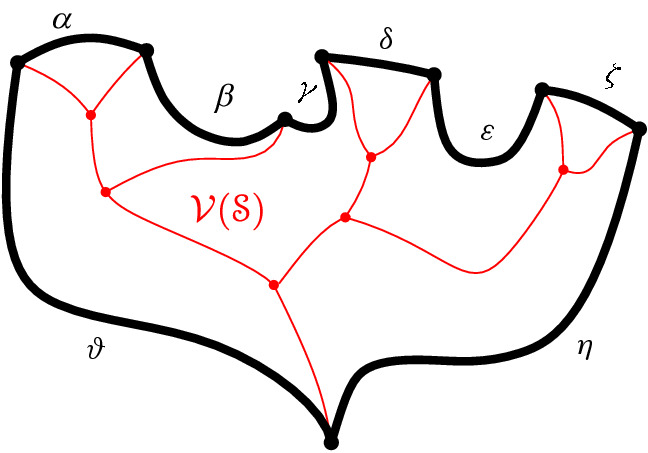
Illustration of  in bold (black) and  in red;

We interpret the core arcs in  as sites that induce a Voronoi diagram  such that , see Fig. 7. By Lemma 2.1, each face of  is incident to exactly one core arc in ; thus, it can be interpreted as the Voronoi region of its incident core arc. Then,  can be viewed as the Voronoi diagram of the arcs in .

The arrangement of a bisector set $\mathcal {J}'\subseteq \mathcal {J}$ is denoted by $\mathcal {A}(\mathcal {J}')$. A *path* *P* in the arrangement $\mathcal {A}(\mathcal {J}')$ is a connected sequence of alternating edges and vertices in this arrangement. An *arc* $\alpha$ of *P* (denoted as $\alpha \in P$) is a maximally connected collection of consecutive edges and vertices of the arrangement along *P* that belong to the same bisector. The common endpoint of two consecutive arcs of *P* is a *vertex of* *P*. An arc of *P* is also called an *edge*. Any two consecutive arcs in *P* are pieces of different bisectors.

Consider the arrangement of a set of *p*-related bisectors $\mathcal {J}_{p,S'}$, $S'\subseteq S$. Since it may consist of several connected components, we also include $\varGamma$ in this arrangement to unify the various components, deriving $\mathcal {A}(\mathcal {J}_{p,S'}\cup \{\varGamma \})$.

### Definition 3.1

**Fig. 8:**
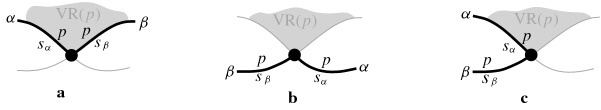
**a** Arcs $\alpha , \beta$ fulfill the *p*-monotone path condition; they do not fulfill it in **b** and **c**

**Fig. 9:**
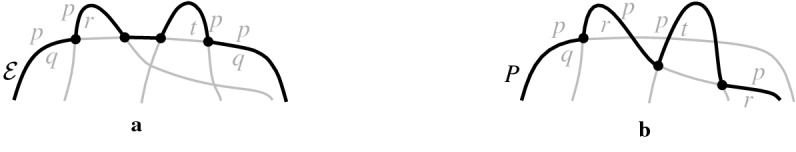
*p*-monotone paths in ${\mathcal {J}}_{p,\{q,r,t\}}$. **a** illustrates the envelope $\mathcal {E}$ of ${\mathcal {J}}_{p,\{q,r,t\}}$

A path in the arrangement of *p*-related bisectors $\mathcal {J}_{p,S'}\cup \{\varGamma \}$, $S'\subseteq S$, is called *p*-*monotone* (or simply *monotone*) if any two consecutive arcs $\alpha ,\beta$ on this path, where $\alpha \subseteq J(p,s_\alpha )$ and $\beta \subseteq J(p,s_\beta )$, coincide (within a neighborhood of their common endpoint) with two Voronoi edges of $\partial {{\,\textrm{VR}\,}}(p,\{p,s_\alpha ,s_\beta \})$ (see Figs. 8, 9).

The boundary of the Voronoi region ${{\,\textrm{VR}\,}}(p,S' \cup \{p\})\cap D_\varGamma$, $S'\subseteq S$, is an example of such a *p*-monotone path, which is called the *envelope* of $\mathcal {J}_{p,S'} \cup \{\varGamma \}$. Figure 9 illustrates examples of *p*-monotone paths, where the envelope is shown in Fig. 9a.

### Definition 3.2

Consider  and let  be the sites in $S\setminus \{s\}$ that define the arcs in . A *boundary curve* $\mathcal {P}$ for  is a closed *s*-monotone path in the arrangement of *s*-related bisectors $\mathcal {J}_{s,S'} \cup \{\varGamma \}$ such that all arcs in  are contained in $\mathcal {P}$. The open portion of the plane enclosed by $\mathcal {P}$ is called the *domain* of $\mathcal {P}$, denoted $D_{\mathcal {P}}$. Given $\mathcal {P}$, let $S_\mathcal {P}=S'$.

**Fig. 10:**
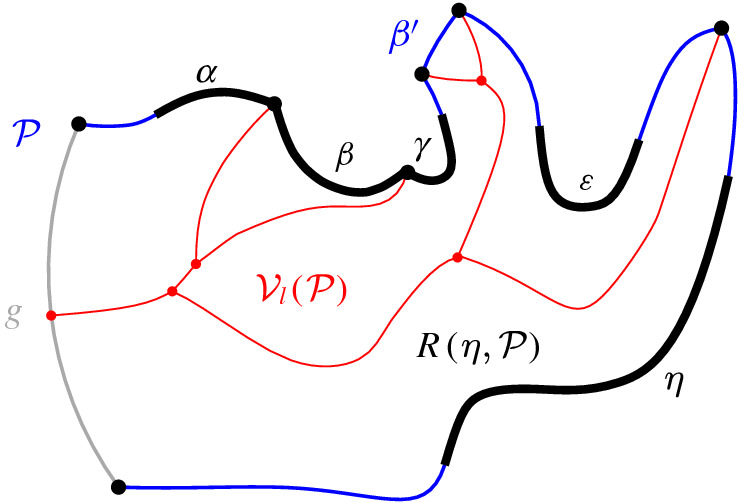
A boundary curve $\mathcal {P}$ on , where the core arcs in  are shown in bold, and its Voronoi-like diagram $\mathcal {V}_l(\mathcal {P})$ is shown in red. The gray arc *g* is a $\varGamma$-arc, and the blue arc $\beta '$ is an auxiliary arc; the remaining arcs are original. The set of core arcs  is shown in Fig. 7

A set  can admit several different boundary curves, see e.g., the different *p*-monotone paths in Fig. 9. One such boundary curve is the boundary of ${{\,\textrm{VR}\,}}(s,S' \cup \{s\})\cap D_\varGamma$, which is called the *envelope* of , $\mathcal {E}= \partial {{\,\textrm{VR}\,}}(s,S' \cup \{s\})\cap D_\varGamma$. The full set  can have only one boundary curve, which is the boundary of ${{\,\textrm{VR}\,}}(s,S) \cap D_\varGamma$. Recall that $\mathscr {S}$ is ordered according to $\partial {{\,\textrm{VR}\,}}(s,S)$, and the same ordering applies to any subset (eqiv. subsequence) $\mathscr {S}'\subset \mathscr {S}$. Figure 10 illustrates a boundary curve for a subset of core arcs from Fig. 7.

A boundary curve $\mathcal {P}$ on  consists of pieces of *s*-related bisectors called *boundary arcs*, and pieces of $\varGamma$, called $\varGamma$-*arcs*. $\varGamma$-arcs correspond to openings of the domain $D_\mathcal {P}$ to infinity. Among the boundary arcs, those containing a core arc of  are called *original* and others, which contain no core arc, are called *auxiliary*. Original boundary arcs in $\mathcal {P}$ are expanded versions of the core arcs in . To distinguish between an original arc $\alpha$ and its core sub-arc in , we use an $^*$ to denote the latter. Figure 10 illustrates a boundary curve $\mathcal {P}$ on  consisting of five original arcs, one auxiliary arc (arc $\beta '$) and one $\varGamma$-arc (arc *g*); the core arcs are illustrated in bold and the set  is shown in Fig. 7. Let $|\mathcal {P}|$ denote the number of boundary arcs in $\mathcal {P}$.

We now define the *Voronoi-like diagram* of a boundary curve $\mathcal {P}$ on . Recall that $S'=\{s_{\alpha } \in S\setminus \{s\} \, | \, \alpha \in \mathscr {S}'\}$ is the set of sites in $S\setminus \{s\}$, which define the core arcs in $\mathscr {S}'$.

### Definition 3.3

Given a boundary curve ${\mathcal {P}}$ on , the *Voronoi-like diagram* of $\mathcal {P}$, denoted $\mathcal {V}_l(\mathcal {P})$, is a plane graph defined on the arrangement of the bisector system $\mathcal {J}_{s,S'}$ that subdivides the domain $D_{\mathcal {P}}$ as follows (see Fig. 10):

- for each boundary arc $\alpha \in \mathcal {P}\setminus \varGamma$, there is exactly one distinct face $R(\alpha ,\mathcal {P})$, whose boundary is an $s_\alpha$-monotone path in $\mathcal {J}_{s_\alpha ,S'} \cup \varGamma$, plus arc $\alpha$;
- the faces cover the domain $D_{\mathcal {P}}$: $\bigcup _{\alpha \in {\mathcal {P}\setminus \varGamma }}\overline{R(\alpha ,\mathcal {P})}=\overline{D_\mathcal {P}}$.

If the boundary curve $\mathcal {P}$ coincides with the envelope $\mathcal {E}=\partial {{\,\textrm{VR}\,}}(s,S' \cup \{s\})\cap D_\varGamma$, then $\mathcal {V}_l(\mathcal {P})$ is the ordinary Voronoi diagram of $S'$ as truncated within the domain of $\mathcal {E}$. That is, $\mathcal {V}_l(\mathcal {P}) = \mathcal {V}_l(\mathcal {E})= \mathcal {V}(S')\cap D_{\mathcal {E}}$ (see Lemma 3.4 and Corollary 3.5 in the sequel). For an arbitrary boundary curve $\mathcal {P}$, the Voronoi-like regions in $\mathcal {V}_l(\mathcal {P})$ are related to the real Voronoi regions in $\mathcal {V}(S')\cap D_{\mathcal {E}}$ as supersets (see the following lemma).

Let $\mathcal {V}(\mathcal {E})=\mathcal {V}(S')\cap D_{\mathcal {E}}$. Any face of the Voronoi diagram $\mathcal {V}(\mathcal {E})$ incident to a boundary arc $\alpha \in \mathcal {E}$ is regarded as the Voronoi region ${{\,\textrm{VR}\,}}(\alpha ,\mathcal {E})$. We show that $R(\alpha ,\mathcal {E})= {{\,\textrm{VR}\,}}(\alpha ,\mathcal {E})$, thus, $\mathcal {V}(\mathcal {E})=\mathcal {V}_l(\mathcal {E})$.

### Lemma 3.4

Let $\mathcal {P}$ be a boundary curve on  and let $\mathcal {E}$ be the envelope of , $\mathcal {E}=\partial {{\,\textrm{VR}\,}}(s,S' \cup \{s\})\cap D_\varGamma$. Let $\alpha \in \mathcal {P}$ and $\tilde{\alpha }\in \mathcal {E}$ be two overlapping arcs where $\alpha ,\tilde{\alpha }\subseteq J(s,s_\alpha )$. Then, $R(\alpha ,\mathcal {P}) \supseteq {{\,\textrm{VR}\,}}(\tilde{\alpha },\mathcal {E})$. Further, if $\alpha$ and $\tilde{\alpha }$ are original, i.e., $\alpha \supseteq \tilde{\alpha }\supseteq \alpha ^*$, where , then .

### Proof

By the definition of a boundary curve, it holds that $\alpha \supseteq \tilde{\alpha }$. By the definition of a Voronoi region, bisector $J(s_\alpha ,\,{\cdot }\,)$ cannot appear in the interior of any Voronoi region in $\mathcal {V}(S')\cap D_\mathcal {E}=\mathcal {V}(\mathcal {E})$. Since $\alpha \supseteq \tilde{\alpha }$, by the definition of a Voronoi-like region, it follows that $R(\alpha ,\mathcal {P}) \supseteq {{\,\textrm{VR}\,}}(\tilde{\alpha },\mathcal {E})$. Suppose that $\alpha$ and $\tilde{\alpha }$ are original; since $S' \subseteq S$, by the monotonicity property of Voronoi regions, we have . $\square$

As an example, refer to the Voronoi-like diagram $\mathcal {V}_l(\mathcal {P})$ of Fig. 10 versus the Voronoi diagram  in Fig. 7: the Voronoi-like region $R(\eta ,\mathcal {P})$ is a superset of the Voronoi region  in Fig. 7; similarly .

Another implication of Lemma 3.4 is that the adjacencies of the Voronoi diagram $\mathcal {V}(\mathcal {E})$, among the original arcs of $\mathcal {E}$, are all preserved in $\mathcal {V}_l(\mathcal {P})$ (see Figs. 7, 10). If $\mathcal {P}=\mathcal {E}$, then $\mathcal {V}_l(\mathcal {E})$ and $\mathcal {V}(\mathcal {E})$ coincide as a direct consequence of Lemma 3.4.

### Corollary 3.5

$\mathcal {V}_l(\mathcal {E})=\mathcal {V}(S')\cap D_\mathcal {E}=\mathcal {V}(\mathcal {E})$ for the envelope $\mathcal {E}$ of .

In the remainder of this section we give basic properties of Voronoi-like regions involving their interaction with the bisectors in $\mathcal {J}$, which we later use in subsequent sections to derive correctness and establish that the Voronoi-like diagram is well defined.

### Properties of Voronoi-Like Regions

The following property establishes that a Voronoi-like region $R(\alpha ,\mathcal {P})$ cannot be intersected by $J(s,s_\alpha )$.

#### Lemma 3.6

For any arc $\alpha \in \mathcal {P}$, $R(\alpha ,\mathcal {P}) \subseteq D(s,s_\alpha )$.

#### Proof

The contrary would yield a forbidden $s_\alpha$-inverse cycle defined by a component of $J(s,s_\alpha )\cap R(\alpha ,\mathcal {P})$ and the incident portion of $\partial R(\alpha ,\mathcal {P})$. $\square$

#### Lemma 3.7

For a boundary curve $\mathcal {P}$, its domain $\overline{D_\mathcal {P}}$ may not contain a *p*-cycle formed by the bisectors of $\mathcal {J}_{s,S_\mathcal {P}} \cup \{\varGamma \}$ for any site $p\in S_\mathcal {P}$.

#### Proof

Let $p\in S_\mathcal {P}$. Any original arc of *p* in $\mathcal {P}$ is bounding ${{\,\textrm{VR}\,}}(p, S_\mathcal {P}\cup \{s\})$, thus, it must have a portion within the interior of ${{\,\textrm{VR}\,}}(p, S_\mathcal {P})$ in $\mathcal {V}(S_\mathcal {P})$. Hence, ${{\,\textrm{VR}\,}}(p,S_\mathcal {P})$ must have some non-empty portion outside the closure of $D_\mathcal {P}$. However, ${{\,\textrm{VR}\,}}(p,S_\mathcal {P})\cap D_\varGamma$ must be enclosed within any *p*-cycle of $\mathcal {J}_{s,S_\mathcal {P}} \cup \{\varGamma \}$, by its definition. Thus, no such *p*-cycle can be contained in $\overline{D_\mathcal {P}}$. $\square$

**Fig. 11:**
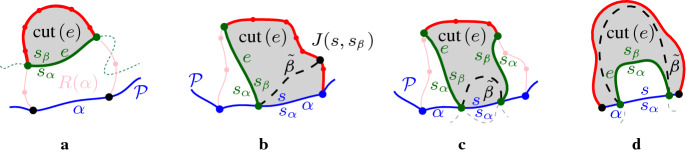
Various cases of Lemma 3.8. The shaded region illustrates $cut (e)\subseteq D(s_\beta ,s_\alpha )$

Next, we give a key property of a Voronoi-like region $R(\alpha ,\mathcal {P})$, called the *cut property*, see Fig. 11. Consider a connected component *e* of $J(s_\alpha ,s_\beta )\cap R(\alpha ,\mathcal {P})$ and let $cut (e)$ denote the portion of region $R(\alpha ,\mathcal {P})$ that is *cut out by e*, as shown shaded in Fig. 11, and defined as follows. If *e* does not intersect $\alpha$, let $cut (e)$ be the portion of the region at the opposite side of *e* as $\alpha$, see Fig. 11a. If *e* is the only component of $J(s_\alpha ,s_\beta )\cap R(\alpha ,\mathcal {P})$ incident to $\alpha$, let $cut (e)$ be the portion of $R(\alpha ,\mathcal {P})$ incident to the side of *e* labeled $s_\beta$, see Fig. 11, b and d. If two different components of $J(s_\alpha ,s_\beta )\cap R(\alpha ,\mathcal {P})$ are incident to $\alpha$, let $cut (e)$ be the portion of $R(\alpha ,\mathcal {P})$ between these two components, see Fig. 11c. Note that if $\beta \in \mathcal {P}$ then only the cases (a) and (b) are possible. On the other hand, if $\mathcal {P}=\mathcal {E}$, and $\alpha ,\beta \in \mathcal {E}$, then $J(s_\alpha ,s_\beta )$ cannot intersect ${{\,\textrm{VR}\,}}(\alpha ,\mathcal {E})$, thus, none of these cases is possible.

#### Lemma 3.8

Suppose bisector $J(s_\alpha ,s_\beta )$ intersects $R(\alpha ,\mathcal {P})$ (see Fig. 11). For any connected component *e* of $J(s_\alpha ,s_\beta )\cap R(\alpha ,\mathcal {P})$, it holds $cut (e)\subseteq D(s_\beta ,s_\alpha )$.

#### Proof

Suppose first that a component *e* of $J(s_\alpha ,s_\beta )\cap R(\alpha ,\mathcal {P})$ does not intersect $\alpha$, see Fig. 11a. Then the label $s_\alpha$ must appear on the same side of *e* as $\alpha$, because otherwise, $\partial cut (e)$ would be an $s_\alpha$-cycle, contradicting Lemma 3.7.

Suppose now that *e* intersects $\alpha$. Then there is a component $\tilde{\beta }$ of $J(s,s_\beta )\cap R(\alpha ,\mathcal {P})$, incident to the intersection point of *e* and $\alpha$, that is contained in $cut (e)$. Since *s*-bisectors can intersect at most twice, it follows that $\tilde{\beta }$ may have both its endpoints on $\alpha$ only if $\beta \notin \mathcal {P}$, because otherwise, $J(s,s_\beta )$ and $J(s,s_\alpha )$ would intersect more than twice. Thus, if $\beta \in \mathcal {P}$, *e* may only have one endpoint on $\alpha$, and no other component of $J(s_\alpha ,s_\beta )\cap R(\alpha ,\mathcal {P})$ may be incident to $\alpha$, see Fig. 11b. Otherwise, $J(s_\alpha ,s_\beta )$ may intersect $\alpha$ twice, resulting in cases (c) or (d) of Fig. 11. No other cases exist.

Consider an arbitrary component *e* of $J(s_\alpha ,s_\beta )\cap R(\alpha ,\mathcal {P})$. Suppose for the sake of contradiction that $cut (e) \not \subseteq D(s_\beta ,s_\alpha )$. Then $J(s_\beta ,s_\alpha )$ must intersect the interior of $cut (e)$ with a component $e'$ of $J(s_\beta ,s_\alpha ) \cap R(\alpha ,\mathcal {P})$, $e'\ne e$. Among any such component, let $e'$ be the first one following *e* in the direction away from $\alpha$. Since $e'$ cannot intersect *e* nor can it intersect $\alpha$, it follows that $e'$ must create an $s_\alpha$-cycle with $\partial cut (e)$, contradicting Lemma 3.7. Figure 17 illustrates such a forbidden $s_\gamma$-cycle created by a piece of $J(s_\beta , s_\gamma )$, shown in dashed lines, and $\partial R(\gamma ,\mathcal {P})$. $\square$

Lemma 3.8 implies that any components of $J(s_\alpha ,s_\beta )\cap R(\alpha ,\mathcal {P})$ must appear sequentially along $\partial R(\alpha ,\mathcal {P})$. That is, in a traversal of $\partial R(\alpha ,\mathcal {P})$, starting at $\alpha$, no component of $J(s_\alpha ,s_\beta )\cap R(\alpha ,\mathcal {P})$ may appear between the endpoints of another. Further, if $J(s_\alpha ,s_\beta )$ intersects $R(\alpha ,\mathcal {P})$, then $J(s,s_\beta )$ must also intersect the domain $D_\mathcal {P}$. We use this fact to establish that $\mathcal {V}_l(\mathcal {P})$ is unique in the following theorem; the proof is deferred to Sect. 5.

#### Theorem 3.9

Given a boundary curve $\mathcal {P}$ of , $\mathcal {V}_l(\mathcal {P})$ is unique, assuming it exists.

The complexity of $\mathcal {V}_l(\mathcal {P})$ is $O(|\mathcal {P}|)$ as it is a planar acyclic graph with exactly one face per boundary arc and vertices of degree 3 (or 1).

## Insertion in a Voronoi-Like Diagram

Consider a boundary curve $\mathcal {P}$ on a set of core arcs  and its Voronoi-like diagram $\mathcal {V}_l(\mathcal {P})$. Let $\beta ^*$ be a core arc in . We define an insertion operation $\oplus$, which adds $\beta ^*$ to $\mathcal {P}$, and derives the boundary curve $\mathcal {P}_\beta = \mathcal {P}\oplus \beta ^*$ and its Voronoi-like diagram $\mathcal {V}_l(\mathcal {P}_\beta )=\mathcal {V}_l(\mathcal {P}) \oplus \beta ^*$. Since $\beta ^*$ is a core arc, it must be entirely contained in the closure of the domain $D_\mathcal {P}$.

**Fig. 12:**
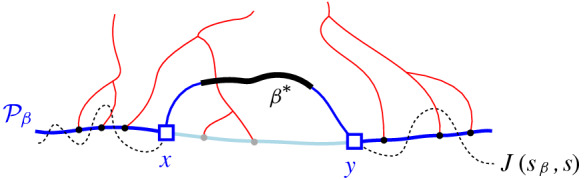
$\mathcal {P}_\beta = \mathcal {P}\oplus \beta$, core arc $\beta ^*$ is bold, black. Endpoints of $\beta$ are *x*, *y*

**Fig. 13:**
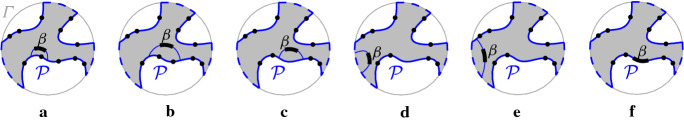
Insertion cases for an arc $\beta$

Given $\mathcal {P}$ and $\beta ^*$, let $\beta \supseteq \beta ^*$ be the connected component of $J(s,s_\beta )\cap \overline{D_{\mathcal {P}}}$ that contains $\beta ^*$ (see Fig. 12). $\mathcal {P}_\beta$ is the boundary curve derived from $\mathcal {P}$ by substituting its portion between the endpoints of $\beta$, with $\beta$ itself. We say that $\mathcal {P}_\beta$ is derived from $\mathcal {P}$ by *inserting* the core arc $\beta ^*$, or equivalently, by inserting the original arc $\beta$. The insertion operation performs the following tasks algorithmically:

- Insert the core arc $\beta ^*$ in $\mathcal {P}$, deriving $\mathcal {P}_\beta = \mathcal {P}\oplus \beta ^*=\mathcal {P}\oplus \beta$. The various cases are illustrated in Fig. 13, see Observation 4.1 below.
- Compute the *merge curve* $J(\beta )$, which defines the boundary of $R(\beta , \mathcal {P}_\beta )$.
- Update $\mathcal {V}_l(\mathcal {P})$, by inserting $J(\beta )$ and deleting any portion of the diagram enclosed by it, to derive $\mathcal {V}_l(\mathcal {P}_\beta )=\mathcal {V}_l(\mathcal {P})\oplus \beta$.

These tasks are standard in relation to site insertion in any Voronoi diagram. We prove their correctness in a Voronoi-like structure, see Theorems 4.3 and 4.4.

### Observation 4.1

All possible cases of inserting arc $\beta ^*\subseteq \beta$ in $\mathcal {P}$ are enumerated as follows (see Fig. 13).

- Arc $\beta$ straddles the endpoint of two consecutive boundary arcs; no arcs in $\mathcal {P}$ are deleted.
- Auxiliary arcs in $\mathcal {P}$ are deleted by $\beta$; their regions are also deleted from $\mathcal {V}_l(\mathcal {P}_\beta )$.
- An arc $\alpha \in \mathcal {P}$ is split into two arcs by $\beta$; $R(\alpha ,\mathcal {P})$ will also be split in two parts.
- A $\varGamma$-arc is split in two by $\beta$; $\mathcal {V}_l(\mathcal {P}_\beta )$ may switch from being a tree to being a forest.
- A $\varGamma$-arc is deleted or shrunk by inserting $\beta$. $\mathcal {V}_l(\mathcal {P}_\beta )$ may become a tree.
- $\mathcal {P}$ already contains a boundary arc ${\bar{\beta }}\supseteq \beta ^*$; then $\beta ={\bar{\beta }}$ and $\mathcal {P}_\beta =\mathcal {P}$.

In terms of auxiliary arcs, $\mathcal {P}_\beta$ may contain fewer, the same number, or even one additional auxiliary arc as compared to $\mathcal {P}$.

**Fig. 14:**
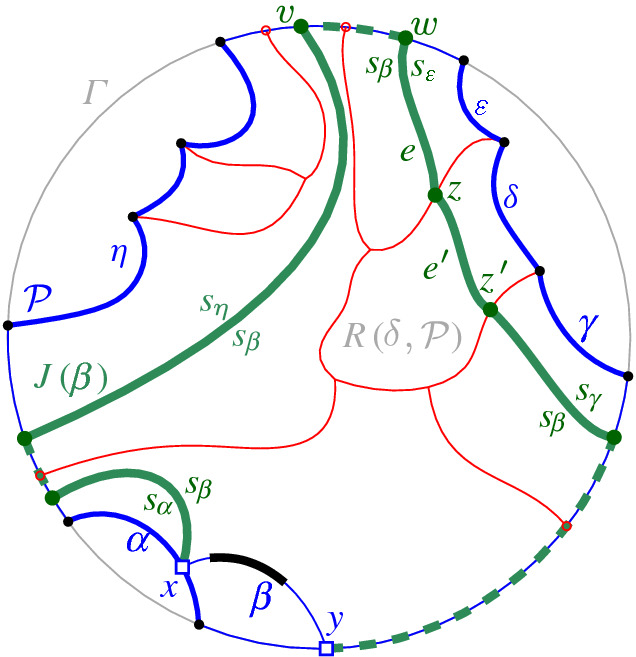
The merge curve $J(\beta )$ (thick, green) on $\mathcal {V}_l(\mathcal {P})$ (thin, red)

Given $\mathcal {V}_l(\mathcal {P})$ and arc $\beta$, we define a *merge curve* $J(\beta )$, which delimits the boundary of $R(\beta , \mathcal {P}_\beta )$. We define $J(\beta )$ algorithmically (see Def. 4.2), starting at an endpoint of $\beta$, and tracing $s_\beta$-related bisectors within the faces of $\mathcal {V}_l(\mathcal {P})$, refer to Fig. 14. We prove that $J(\beta )$ is indeed an $s_\beta$-monotone path that connects the endpoints of $\beta$ (Theorem 4.3). Let *x*, *y* denote the endpoints of $\beta$, where $x\beta y$ appear in counterclockwise order. We assume a counterclockwise traversal of $\mathcal {P}$. Refer to Fig. 14.

### Definition 4.2

Given $\mathcal {V}_l(\mathcal {P})$ and arc $\beta \subseteq J(s,s_\beta )$, the *merge curve* $J(\beta )$ is a path $(v_1,\dots ,v_m)$ in the arrangement of $s_\beta$-related bisectors, $\mathcal {A}(\mathcal {J}_{s_\beta ,S_\mathcal {P}} \cup \{\varGamma \})$, connecting the endpoints of $\beta$, $v_1=x$ and $v_m=y$. Each edge $e_i=(v_i,v_{i+1})$ is an arc of a bisector $J(s_\beta ,\,{\cdot }\,)$, called a *bisector* edge, or an arc on $\varGamma$. We assume a clockwise ordering of $J(\beta )$. For $i=1$: if $x \in J(s_\beta , s_{\alpha })$, then $e_1 \subseteq J(s_\beta ,s_{\alpha })$; if $x \in \varGamma$, then $e_1 \subseteq \varGamma$. Given $v_i$, vertex $v_{i+1}$ and edge $e_{i+1}$ are defined as follows.

(i) If $e_{i} \subseteq J(s_\beta , s_{\alpha })$, let $v_{i+1}$ be the other endpoint of the connected component of $J(s_\beta ,s_{\alpha })\cap R(\alpha ,\mathcal {P})$ incident to $v_{i}$. If $v_{i+1}\in J(s_\beta ,\,{\cdot }\,)\cap J(s_\beta , s_{\alpha })$, then $e_{i+1}\subseteq J(s_\beta ,\,{\cdot }\,)$. If $v_{i+1}\in \varGamma$, then $e_{i+1} \subseteq \varGamma$. (In Fig. 14, see $e_{i}=e',v_{i}=z,v_{i+1} = z'$.)
(ii) If $e_{i} \subseteq \varGamma$, let *g* be the $\varGamma$-arc in $\mathcal {P}$ incident to $v_i$, in clockwise order. Let $e_{i+1} \subseteq J(s_\beta ,s_\gamma )$, where $\gamma \in \mathcal {P}$ and $R(\gamma ,\mathcal {P})$ is the first region, incident to *g* clockwise from $v_{i}$ such that $J(s_\beta , s_\gamma )$ intersects $g \cap \overline{R(\gamma ,\mathcal {P})}$; let $v_{i+1}$ be this intersection point. (In Fig. 14, see $v_i = v$ and $v_{i+1} = w$.)

The following theorem shows that $J(\beta )$ forms an $s_{\beta }$-monotone path joining the endpoints of $\beta$. We defer its proof to the end of this section (Sect. 4.1).

**Fig. 15:**
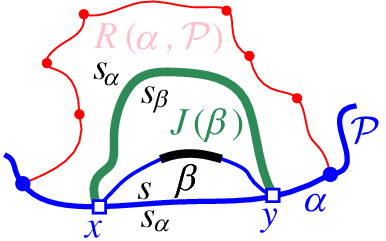
If $\beta$ splits $\alpha$, $J(\beta ) \subset R(\alpha ,\mathcal {P})$ would yield a forbidden $s_\alpha$-inverse cycle

**Fig. 16:**
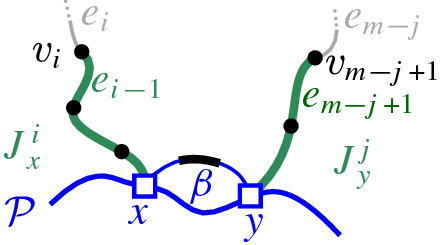
$J_x^i$ and $J_y^j$ in Sect. 4.1

### Theorem 4.3

The merge curve $J(\beta )$ is a unique $s_{\beta }$-monotone path in the arrangement of $s_\beta$-related bisectors $\mathcal {A}(\mathcal {J}_{s_\beta ,S_\mathcal {P}} \cup \varGamma )$ connecting the endpoints of $\beta$. Further:

- If arc $\beta$ splits a single arc $\alpha \in \mathcal {P}$ (case (c) of Observation 4.1) then $J(\beta )$ must intersect $R(\alpha ,\mathcal {P})$ in two different components, $e_1,e_{m-1} \subseteq J(s_\alpha ,s_\beta )$. $J(\beta )$ can intersect any other region in $\mathcal {V}_l(\mathcal {P})$ at most once.
- $J(\beta )$ cannot intersect the region of any arc in $\mathcal {P}\setminus \mathcal {P}_\beta$, which gets deleted by the insertion of $\beta$, nor can it intersect arc $\beta$ in its interior.

Let $T(\beta )$ denote the portion of $\mathcal {V}_l(\mathcal {P})$ enclosed by $J(\beta )$ and $\mathcal {P}\setminus \mathcal {P}_\beta$. Let $\mathcal {V}_l(\mathcal {P})\oplus \beta$ denote the graph obtained from $\mathcal {V}_l(\mathcal {P})$ by deleting $T(\beta )$ and substituting it with $J(\beta )$, i.e., $\mathcal {V}_l(\mathcal {P})\oplus \beta =(\mathcal {V}_l(\mathcal {P})\setminus T(\beta ))\cup J(\beta ),$

### Theorem 4.4

${\mathcal {V}_l(\mathcal {P})\oplus \beta }$ is the Voronoi-like diagram $\mathcal {V}_l(\mathcal {P}_\beta )$.

### Proof

By construction, ${\mathcal {V}_l(\mathcal {P})\oplus \beta }$ induces a subdivision of the domain $D_{\mathcal {P}_\beta }$. By Theorem 4.3, $J(\beta )$, and thus, $\partial R(\beta )\setminus \beta$, is an $s_\beta$-monotone path connecting the endpoints of $\beta$. For any arc $\alpha \in \mathcal {P}$ such that $J(\beta )$ passes through $R(\alpha ,\mathcal {P})$, the boundary of the updated face in ${\mathcal {V}_l(\mathcal {P})\oplus \beta }$ remains an $s_\alpha$-monotone path, by the definition of $J(\beta )$. Thus, for any face *f* of ${\mathcal {V}_l(\mathcal {P})\oplus \beta }$ incident to an arc $\alpha \ne \beta$, its boundary $\partial f \setminus \alpha$ is an $s_\alpha$-monotone path, hence, it satisfies the first requirement of Definition 3.3.

Since $J(\beta )$ can enter any region in $\mathcal {V}_l(\mathcal {P})$ at most once (except from case (c) of Observation 4.1) it cannot create a face that may remain in the interior of $D_\mathcal {P}$. Further, $J(\beta )$ cannot pass through any region of an arc in $\mathcal {P}\setminus \mathcal {P}_\beta$, thus, such a region must be enclosed by $J(\beta )$ and will be deleted. Hence, any face of ${\mathcal {V}_l(\mathcal {P})\oplus \beta }$ must be incident to a boundary arc of $\mathcal {P}_\beta$, satisfying also the second requirement of Definition 3.3. Since, by Theorem 3.9, the Voronoi-like diagram of a boundary curve is unique, it follows that ${\mathcal {V}_l(\mathcal {P})\oplus \beta }=\mathcal {V}_l(\mathcal {P}_\beta )$. $\square$

**Fig. 17:**
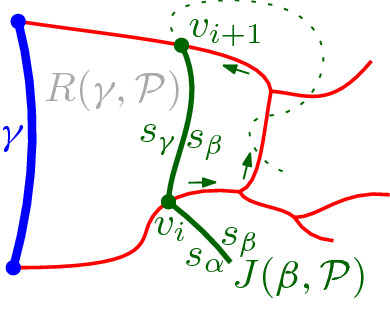
Impossible configuration of $J(s_\beta , s_\gamma )$. Scanning $\partial R(\gamma ,\mathcal {P})$ from $v_i$ counterclockwise, Lemma 3.8 assures that $v_{i+1}$ is the first encountered intersection of $J(s_\beta , s_\gamma )$ with $\partial R(\gamma ,\mathcal {P})$

The tracing of the merge curve $J(\beta )$ within $\mathcal {V}_l(\mathcal {P})$ can be performed similarly to any ordinary Voronoi diagram (see, e.g., [2, Ch. 7.5.3]). This is correct in a Voronoi-like diagram as a result of the cut property of Lemma 3.8: when $J(\beta )$ enters a region $R(\gamma ,\mathcal {P})$ at a point $v_i$, we can determine $v_{i+1}$ by scanning $\partial R(\gamma ,\mathcal {P})$ counterclockwise sequentially, until we encounter the first intersection with $J(s_\beta , s_\gamma )$. Lemma 3.8 assures that no intersection of $J(s_\beta , s_\gamma )$ with $\partial R(\gamma ,\mathcal {P})$ between $v_i$ and $v_{i+1}$ is possible, such as the one shown in Fig. 17. Thus, we can state the following fact.

### Lemma 4.5

Let $e_i=(v_i,v_{i+1})$ be an edge of $J(\beta )$ in $R(\gamma ,\mathcal {P})$. Given $v_i$, we can determine $v_{i+1}$ by sequentially scanning $\partial R(\gamma ,\mathcal {P})$ counterclockwise from $v_i$ (i.e., away from $\gamma$) until the first intersection of $J(s_\beta , s_\gamma )$ with $\partial R(\gamma ,\mathcal {P})$ which determines $v_{i+1}$.

Special care is required in cases (c), (d), and (e) of Observation 4.1 to identify the first edge of $J(\beta )$, as $\beta$ does not overlap any feature of $\mathcal {V}_l(\mathcal {P})$ in these cases. To handle them we need to define some additional parameters.

Let ${\tilde{\mathcal {P}}}$ denote the finer version of ${\mathcal {P}}$ derived by intersecting its $\varGamma$-arcs with $\mathcal {V}_l(\mathcal {P})$, i.e., partitioning the $\varGamma$-arcs of $\mathcal {P}$ into finer pieces by the incident faces of $\mathcal {V}_l(\mathcal {P})$. Since the complexity of $\mathcal {V}_l(\mathcal {P})$ is $O(|\mathcal {P}|)$, it follows that $|{\tilde{\mathcal {P}}}|$ is also $O(|\mathcal {P}|)$.

### Definition 4.6

Let $\alpha$ and $\gamma$ denote the original arcs preceding and following $\beta$ on $\mathcal {P}_\beta$. We assume a counterclockwise traversal of $\mathcal {P}$ and $\mathcal {P}_\beta$.

(i) Let $d_1(\beta ,\mathcal {P}_\beta )$ denote the number of auxiliary arcs that appear on $\mathcal {P}_\beta$ from $\alpha$ to $\beta$.
(ii) Let $d_2(\beta ,\mathcal {P}_\beta )$ denote the number of auxiliary arcs that appear on $\mathcal {P}$ between the endpoints of $\beta$ that get deleted by the insertion of $\beta$.
(iii) In case (c) of Observation 4.1, where $\beta$ splits an arc $\omega$ in two arcs $(\omega _1,\omega _2)$, let $r(\beta ,\mathcal {P}_\beta )=\min {\{|\partial R(\omega _1, \mathcal {P}_\beta )|, |\partial R(\omega _2,\mathcal {P}_\beta )|\}}$; in other cases, let $r(\beta ,\mathcal {P}_\beta )=0$.
(iv) In case (d) of Observation 4.1, where $\beta$ splits a $\varGamma$-arc, let ${\tilde{d}}(\beta ,\mathcal {P}_\beta )$ denote the number of fine $\varGamma$-arcs on ${\tilde{\mathcal {P}}}_\beta$ from $\alpha$ to $\beta$ (i.e., the number of regions in $\mathcal {V}_l(\mathcal {P}_\beta )$ incident to $\varGamma$ from $\alpha$ to $\beta$); in all other cases, ${\tilde{d}}(\beta ,\mathcal {P}_\beta ) = 0$.

### Lemma 4.7

Given $\alpha$, $\gamma$, and $\mathcal {V}_l(\mathcal {P})$, the merge curve $J(\beta )$ can be computed in time $O( |J(\beta )|+d_1(\beta ,\mathcal {P}_\beta ) +d_2(\beta ,\mathcal {P}_\beta ) +r(\beta , \mathcal {P}_\beta )+{\tilde{d}}(\beta ,\mathcal {P}_\beta ))$.

### Proof

We assume a counterclockwise (ccw) ordering of $\mathcal {P}$. We first determine the endpoints of $\beta$ in time $O(d_1(\beta ,\mathcal {P}_\beta )+ d_2(\beta ,\mathcal {P}_\beta ))$ by scanning sequentially the arcs in $\mathcal {P}$ starting at $\alpha$ and moving ccw (towards $\gamma$) until the endpoints of $\beta$ are determined. Note that $\beta$ contains the core arc $\beta ^*$, therefore, we can easily identify the correct component of $J(s,s_\beta )\cap D_\mathcal {P}$ during the scan, even if $J(s,s_\beta )$ intersects $\mathcal {P}$ multiple times. This scan also determines which case of Observation 4.1 is relevant.

Let $T(\beta )$ denote the portion of $\mathcal {V}_l(\mathcal {P})$ that is enclosed by $J(\beta )$ and ${\mathcal {P}\setminus \mathcal {P}_\beta }$. $T(\beta )$ gets deleted by the insertion of $\beta$. It is an embedded forest, which by Theorem 4.3 is incident to the following faces of $\mathcal {V}_l(\mathcal {P})$: one face for each bisector edge of $J(\beta )$, and one face for each auxiliary arc $\alpha '\in \mathcal {P}\setminus \mathcal {P}_\beta$. The latter number is counted in $d_2(\beta , \mathcal {P}_\beta )$. We infer that $T(\beta )$ has complexity $O(|J(\beta )| + d_2(\beta , \mathcal {P}_\beta ))$.

To compute $J(\beta )$, we trace $T(\beta )$ in time $O(|T(\beta )|)$, after having identified one of its leaves, as normally done in an ordinary Voronoi diagram. This statement is correct due to Theorem 4.3 and Lemma 4.5. However, we first need to identify one leaf of $T(\beta )$, and certain cases of Observation 4.1 may require additional scans, which can increase the time complexity over $|T(\beta )|$. We give the case analysis in the remainder of this proof.

Suppose first that $T(\beta )$ has a leaf on $\mathcal {P}$. Then, in all cases of Observation 4.1, except cases (d) and (e), a leaf of $T(\beta )$ is identified by the initial scan. In case (e), $\beta$ has at least one endpoint on a boundary arc $\rho$ of $\mathcal {P}$, see Fig. 14; we identify a leaf by scanning ${\tilde{\mathcal {P}}}$ starting at $\rho$ and moving towards the other endpoint of $\beta$. This scan takes only one step as the leaf will be incident to the first $\varGamma$-arc neighboring $\rho$ on ${\tilde{\mathcal {P}}}$. In case (d), both endpoints of $\beta$ are on $\varGamma$. We scan ${\tilde{\mathcal {P}}}$ from $\alpha$ to $\beta$ until we locate the first endpoint *x* of $\beta$. A leaf of $T(\beta )$ must be incident to the fine $\varGamma$-arc that contains *x*. Since all the encountered $\varGamma$-arcs remain in ${\tilde{\mathcal {P}}}_\beta$, the term $O({\tilde{d}}(\beta ,\mathcal {P}_\beta ))$ is added to the overall time complexity.

Suppose now that $T(\beta )$ has no leaf on $\mathcal {P}$. Then $\beta$ is enclosed within a single Voronoi-like region $R(\omega ,\mathcal {P})$. There are three cases to consider: Observation 4.1, (c), (d), and (e).

**Fig. 18:**
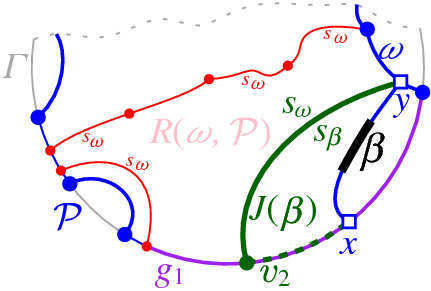
Case (e) of Observation 4.1, where $T(\beta )$ has no leaf on $\mathcal {P}$. Endpoint *x* lies on a fine $\varGamma$-arc $g_1$ bounding $R(\omega ,\mathcal {P})$, and $y\in \omega$

**Fig. 19:**
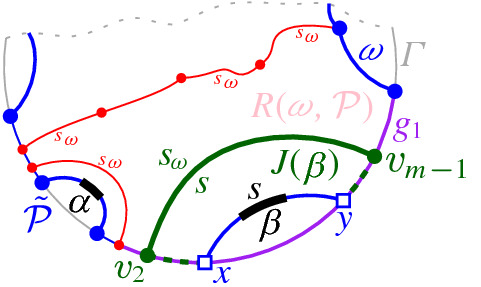
Case (d) of Observation 4.1, where $T(\beta )$ has no leaf on $\mathcal {P}$. Both *x*, *y* lie on a fine $\varGamma$-arc $g_1$ bounding $R(\omega ,\mathcal {P})$

In case Observation 4.1 (c), the insertion of $\beta$ splits arc $\omega$ in two parts, $\omega _1$ and $\omega _2$. We scan $\partial R(\omega ,\mathcal {P})$ sequentially until an intersection with $J(s_{\omega },s_{\beta })$ is found. This intersection point is a leaf of $T(\beta )$ within the domain of $\mathcal {P}$. We start scanning from both endpoints of $\omega$, tracing the shorter among $\partial R(\omega _1,\mathcal {P}_\beta )$ and $\partial R(\omega _2, \mathcal {P}_\beta )$. This adds the term $r(\beta ,\mathcal {P}_\beta )$ to the overall time complexity.

In cases (d) and (e) of Observation 4.1, $J(\beta ) \subseteq R(\omega ,\mathcal {P}) \cup \varGamma$, since otherwise $J(\beta )$ would intersect the region $R(\omega ,\mathcal {P})$ twice, contradicting Theorem 4.3. Thus, $J(\beta )$ consists of a single bisector $J(s_\omega ,s_\beta )$ and one or two $\varGamma$-arcs, see Figs. 18 and 19, respectively. Therefore, we only need to identify $\omega$. In case (e), $\omega$ is identified during the initial scan. In case (d), $\beta$ has both its endpoints on $\varGamma$, and we scan ${\tilde{\mathcal {P}}}$ from $\alpha$ to $\beta$ until we encounter the fine $\varGamma$-arc that contains the first endpoint of $\beta$; the latter $\varGamma$-arc bounds the region $R(\omega ,\mathcal {P})$. This scan adds the term $O({\tilde{d}}(\beta , \mathcal {P}_\beta ))$ to the time complexity. $\square$

### Proving Theorem 4.3

In this section we prove Theorem 4.3. The proof is technical but it is self-contained and it is not necessary for following the rest of the paper. We first establish the following lemma.

#### Lemma 4.8

The merge curve $J(\beta )$ cannot intersect arc $\beta$, other than its endpoints.

#### Proof

Suppose that an edge $e_i$ of $J(\beta )$, such that $e_i\subseteq J(s_\alpha ,s_\beta )$ and $e_i \subseteq R(\alpha ,\mathcal {P})$, intersects arc $\beta$. Then $J(s,s_\alpha )$ must also pass through the same intersection point within $R(\alpha ,\mathcal {P})$. But an *s*-related bisector $J(s,s_\alpha )$ can never intersect $R(\alpha ,\mathcal {P})$, by Lemma 3.6. $\square$

The following observation is used throughout the proofs in this section.

#### Lemma 4.9

For any site $p\in S \setminus \{s\}$, $D(s,p) \cap D_\mathcal {P}$ is connected. Thus, any components of the same *s*-related bisector $J(s,\,{\cdot }\,) \cap D_\mathcal {P}$ must appear along $\mathcal {P}$ sequentially, one after another.

#### Proof

If we assume the contrary, we obtain a forbidden *s*-inverse cycle defined by $J(s,\cdot )$ and $\mathcal {P}$, which contradicts Lemma 2.3. $\square$

We now establish that $J(\beta )$ cannot pass through any region of an auxiliary arc in $\mathcal {P}\setminus \mathcal {P}_\beta$ that gets deleted by the insertion of $\beta$.

#### Lemma 4.10

**Fig. 20:**
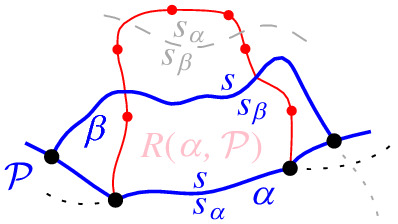
Illustrations for Lemma 4.10

Let $\alpha \in \mathcal {P}$ but $\alpha \not \subseteq \mathcal {P}_\beta$. Then $R(\alpha ,\mathcal {P}) \subset D(s_\beta ,s_{\alpha })$, see Fig. 20.

#### Proof

By Lemma 3.6, it holds that $R(\alpha ,\mathcal {P}) \subseteq D(s,s_\alpha )$. Let $R_s=R(\alpha ,\mathcal {P}) \cap D(s,s_\beta )$ and $R_\beta = R(\alpha ,\mathcal {P}) \cap D(s_\beta ,s)$. By transitivity of dominance regions we have $R_\beta \subseteq D(s_\beta ,s_\alpha )$. By Lemma 4.9, $R_s$ is not incident to $\alpha$. Thus, if $J(s_\beta ,s_\alpha )$ intersected $R_s$ then it would create an $s_\alpha$-cycle with the boundary of $R(\alpha ,\mathcal {P})$, contradicting Lemma 3.7, see the dashed gray line in Fig. 20. This also implies that $R_s \subseteq D(s_\beta ,s_\alpha )$. Thus, $R(\alpha ,\mathcal {P}) = R_s \cup R_\beta \subseteq D(s_\beta ,s_\alpha )$. $\square$

In the following we prove that $J(\beta )$ is an $s_\beta$-monotone path connecting the endpoints of $\beta$. To this aim we perform a bi-directional induction on the vertices of $J(\beta )$.

Let $J_x^i = (v_1, v_2, \ldots , v_i)$, $1\le i < m$, be the subpath of $J(\beta )$ starting at $v_1=x$ up to vertex $v_i$, including a small neighborhood of $e_{i}$ incident to $v_i$, see Fig. 16. Note that vertex $v_i$ uniquely determines $e_{i}$, however, its other endpoint is not yet specified. Similarly, let $J_y^j = (v_m, v_{m-1}, \ldots , v_{m-j+1})$, $1\le j < m$, denote the subpath of $J(\beta )$, starting at $v_m$ up to vertex $v_{m-j+1}$, including a small neighborhood of edge $e_{m-j}$. For any bisector edge $e_\ell \in J(\beta )$, let $\alpha _\ell$ denote the boundary arc that induces $e_\ell$, i.e., $e_\ell \subseteq J(s_{\alpha _\ell },s_\beta ) \cap R(\alpha _\ell ,\mathcal {P})$.

*Inductive hypothesis*:   Suppose $J_x^i$ and $J_y^{j}$, $i,j\ge 1$, are disjoint $s_\beta$-monotone paths. Suppose further that each bisector edge of $J_x^i$ and of $J_y^j$ passes through a distinct region $R(\alpha _\ell ,\mathcal {P})$ in $\mathcal {V}_l(\mathcal {P})$, where $\alpha _\ell$ is distinct for $1\le \ell \le i$ and $m-j\le \ell <m$, except possibly $\alpha _i=\alpha _{m-j}$ and $\alpha _1=\alpha _{m-1}$.

*Inductive step*:   Assuming that $i+j<m$, we prove that at least one of $J_x^i$ or $J_y^j$ can grow to $J_x^{i+1}$ or $J_y^{j+1}$ respectively at a *valid* vertex (Lemmas 4.11, 4.12), entering a new region of $\mathcal {V}_l(\mathcal {P})$ that has not been visited by $J_x^i$ or $J_y^j$ (Lemma 4.14). A vertex is called *valid* if it belongs to $\mathcal {A}(\mathcal {J}_{s_\beta ,S_\mathcal {P}} \cup \{\varGamma \})$ or it is an endpoint of $\beta$. When $i+j=m$, a finish condition is given in Lemma 4.13. The base case for $i=j=1$ is trivially true. In the remaining section we prove correctness of the inductive step.

Suppose that $e_i\subseteq J(s_{\alpha _i},s_\beta )$ and $v_i\in \partial R(\alpha _i,\mathcal {P})$. To show that $v_{i+1}$ is a valid vertex it is enough to show that (1) $v_{i+1}$ cannot be on $\alpha _i$, and (2) if $v_i$ is on a $\varGamma$-arc then $v_{i+1}$ can be determined on the same $\varGamma$-arc. However, we cannot easily derive these conclusions directly. Instead we show that if $v_{i+1}$ is not valid then $v_{m-j}$ will have to be valid. In the following lemmata we assume that the inductive hypothesis holds.

#### Lemma 4.11

Suppose $e_i\subseteq J(s_{\alpha _i},s_\beta )$ but $v_{i+1}\in \alpha _i$, that is, $e_i$ hits arc $\alpha _i\in \mathcal {P}$, and thus, $v_{i+1}$ is not a valid vertex. Then vertex $v_{m-j}$ must be a valid vertex in $\mathcal {A}(\mathcal {J}_{s_\beta ,S_\mathcal {P}})$, and $v_{m-j}$ cannot be on $\mathcal {P}$.

**Fig. 21:**
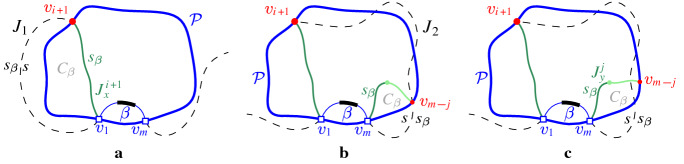
The assumption that edge $e_i = (v_i,v_{i+1})$ of the merge curve $J_x^i$ hits a boundary arc of $\mathcal {P}$ as in Lemma 4.11

#### Proof

Suppose vertex $v_{i+1}$ of $e_{i}$ lies on arc $\alpha _i$ as shown in Fig. 21a. Vertex $v_{i+1}$ is the intersection point of related bisectors $J(s, s_{\alpha _i})$, $J(s_\beta , s_{\alpha _i})$ and thus also of $J(s,s_\beta )$. Thus, $v_1,v_m,v_{i+1} \in J(s,s_\beta )$. By the inductive hypothesis, no other vertex of $J_x^i$ nor $J_y^j$ can be on $J(s,s_\beta )$. Vertices $v_1,v_{i+1},v_m$ appear on $\mathcal {P}$ in clockwise order, because $J_x^{i+1}$ cannot intersect $\beta$. Arc $\beta$ partitions $J(s,s_\beta )$ in two parts: $J_1$ incident to $v_1$ and $J_2$ incident to $v_m$. We claim that $v_{i+1}$ must lie on $J_2$, as otherwise, $J_x^{i+1}$ and $J_1$ would form a forbidden $s_\beta$-inverse cycle, see the dashed black and the green solid curve in Fig. 21a, contradicting Lemma 2.3. This cycle must be $s_\beta$-inverse because $J_x^{i+1} \subseteq \overline{D_\mathcal {P}}$, and all components of $J(s,\,{\cdot }\,) \cap D_\mathcal {P}$ must appear sequentially along $\mathcal {P}$ by Lemma 4.9.

Thus, $v_{i+1}$ lies on $J_2$. Further, by Lemma 4.9, the components of $J_2 \cap D_\mathcal {P}$ appear on $\mathcal {P}$ clockwise after $v_{i+1}$ and before $v_m$, as shown in Fig. 21b, which illustrates $J(s,s_\beta )$ as a black dashed curve.

Now consider $J_y^{j}$. We show that $v_{m-j}$ cannot be on $\mathcal {P}$. First observe that $v_{m-j}$ cannot lie on $\mathcal {P}$, clockwise after $v_m$ and before $v_1$, since $J_y^{j+1}$ cannot cross $\beta$. We prove that $v_{m-j}$ cannot lie on $\mathcal {P}$ clockwise after $v_1$ and before $v_{i+1}$. To see that, note that edge $e_{m-j}$ cannot cross any non-$\varGamma$ edge of $J_x^{i+1}$, because by the inductive hypothesis, $\alpha _{m-j}$ is distinct from all $\alpha _\ell ,\ell \le i$. In addition, by the definition of a $\varGamma$-arc, $v_{m-j}$ cannot lie on any $\varGamma$-arc of $J_x^{i}$. Finally, we show that $v_{m-j}$ cannot lie on $\mathcal {P}$ clockwise after $v_{i+1}$ and before $v_m$. If $v_{m-j}$ lay on the boundary arc $\alpha _{m-j}$ then we would have $v_{m-j}\in J(s,s_\beta )$. This would define an $s_\beta$-inverse cycle $C_\beta$, formed by $J_y^{j+1}$ and $J(s_\beta , s)$, see Fig. 21b, similarly to the first paragraph of this proof. If $v_{m-j}$ lay on a $\varGamma$-arc then there would also be a forbidden $s_\beta$-inverse cycle formed by $J_y^{j+1}$ and $J(s,s_\beta )$ because in order to reach $\varGamma$, edge $e_i$ must cross $J(s,s_\beta )$. See the dashed black and the green curve in Fig. 21c. Thus $v_{m-j}\notin \mathcal {P}$. Since $v_{m-j}\in \partial R(\alpha _{i+1})$ but $v_{m-j}\notin \mathcal {P}$, it must be a vertex of $\mathcal {A}(\mathcal {J}_{s_\beta ,S_\mathcal {P}})$. $\square$

The proof for the following lemma is similar.

#### Lemma 4.12

Suppose vertex $v_i$ is on a $\varGamma$-arc $g\in \mathcal {P}$ but $v_{i+1}$ cannot be determined because no bisector $J(s_\beta , s_\gamma )$ intersects $\overline{R(\gamma ,\mathcal {P})}\cap g$, clockwise from $v_i$. Then vertex $v_{m-j}$ must be a valid vertex in $\mathcal {A}(\mathcal {J}_{s_\beta ,S_\mathcal {P}})$ and $v_{m-j}$ cannot be on $\mathcal {P}$.

**Fig. 22:**
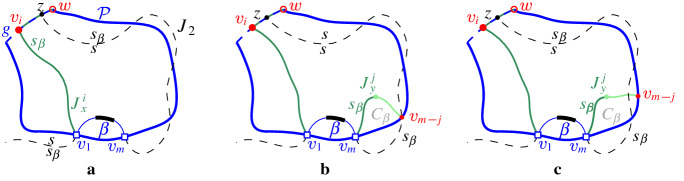
The assumption that $v_i \in \varGamma$ and $v_{i+1}$ of the merge curve $J_x^i$ cannot be determined as in Lemma 4.12

#### Proof

We truncate the $\varGamma$-arc *g* to its portion clockwise from $v_i$; let *w* be the endpoint of *g* clockwise from $v_i$, see Fig. 22a. If no $J(s_\beta , s_\gamma )\cap R(\gamma ,\mathcal {P})$ intersects *g*, as we assume in this lemma, then $R(\gamma ,\mathcal {P})\cap g\subseteq D(s_\beta , s_\gamma )$, for any region $R(\gamma ,\mathcal {P})$ incident to *g*. Thus, $w \in D(s_\beta , s)$. However, $v_i \in D(s,s_\beta )$, since, by Lemma 3.6, $R(\alpha _{i-1})\subseteq D(s,s_{\alpha _{i-1}})$ and $v_i$ is incident to $J(s_\beta ,s_{\alpha _{i-1}})\cap R(\alpha _{i-1})$. Thus, $J(s,s_\beta )$ must intersect *g* at some point *z* clockwise from $v_i$. Arc $\beta$ partitions $J(s,s_\beta )$ in two parts: $J_1$ incident to $v_1$ and $J_2$ incident to $v_m$. Lemma 4.9 implies that all components of $J_2 \cap D_\mathcal {P}$ appear on $\mathcal {P}$ clockwise after $v_{i}$ and before $v_m$, as shown by the black dashed curve in Fig. 22a; also *z* lies on $J_2$.

Now we can show that vertex $v_{m-j}$ of $J_y^{j}$ cannot be on $\mathcal {P}$ analogously to the proof of Lemma 4.11. The only difference is that we must additionally show that $v_{m-j}$ cannot lie on $\mathcal {P}$ clockwise after $v_i$ and before *w*. But this holds already by the assumption in the lemma statement. Refer to Fig. 22, b and c. We conclude that $v_{m-j}$ cannot lie on $\mathcal {P}$ and it is a valid vertex of $\mathcal {A}(\mathcal {J}_{s_\beta ,S_\mathcal {P}})$. $\square$

Lemma 4.13 in the sequel provides a finish condition for the induction, when $J_x^i$ and $J_y^j$ are incident to a common region or to a common $\varGamma$-arc. When it is met, the merge curve $J(\beta )$ is a concatenation of $J_x^i$ and $J_y^j$.

#### Lemma 4.13

Suppose $i+j>2$ and either (1) or (2) holds: (1) $v_i$ and $v_{m-j+1}$ are incident to the same region $R(\alpha _i,\mathcal {P})$ and $e_i,e_{m-j}\subseteq J(s_\beta , s_{\alpha _i})$, i.e., $\alpha _i=\alpha _{m-j}$; or (2) $v_i$ and $v_{m-j+1}$ are on the same $\varGamma$-arc *g* of $\mathcal {P}$ and $e_i, e_{m-j} \subseteq \varGamma$. Then $v_{i+1}=v_{m-j+1}$, $v_{m-j}=v_i$, and $m=i+j$.

**Fig. 23:**
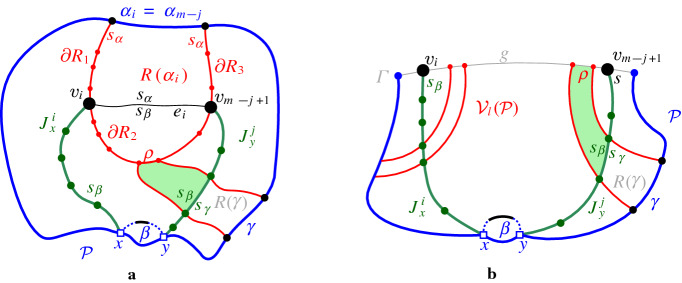
Illustrations for Lemma 4.13. **a** corresponds to condition (1) and **b** to condition (2). The label $R(\gamma )$ abbreviates $R(\gamma , \mathcal {P})$ and the label $R(\alpha _i)$ abbreviates $R(\alpha _i,\mathcal {P})$

#### Proof

Let $\alpha = \alpha _i$. Suppose (1) holds, then $e_i,e_{m-j}\subseteq J(s_\beta , s_{\alpha })$, see Fig. 23a. The boundary $\partial R(\alpha _i,\mathcal {P})$ is partitioned in four parts, using a counterclockwise traversal starting at $\alpha _{i}$: 1. $\partial R_1$, from the endpoint of arc $\alpha _i$ to $v_i$; 2. $\partial R_2$, from $v_i$ to $v_{m-j+1}$; 3. $\partial R_3$, from $v_{m-j+1}$ to the next endpoint of $\alpha _i$; and 4. arc $\alpha _i$. We show that $e_i$ and $e_{m-j}$ cannot hit any of these parts, thus, $e_i=e_{m-j}$.

(i) Edge $e_i$ cannot hit $\partial R_1$ and edge $e_{m-j}$ cannot hit $\partial R_3$, by the cut property of Lemma 3.8.
(ii) We prove that edge $e_i$ cannot hit $\partial R_2$ (analogously for edge $e_{m-j}$). Let $\rho$ be any edge on $\partial R_2$. (If $v_i\in \rho$ or $v_{m-j+1} \in \rho$, assume that $\rho$ is truncated with endpoint $v_i$ or $v_{m-j+1}$ respectively).
  Suppose that $\rho$ is a bisector edge, $\rho \subseteq J(s_\alpha ,s_\gamma )$, see Fig. 23a. Then at least one of $J_y^j$, $J_x^i$, or $\beta$ must pass through $R(\gamma ,\mathcal {P})$. Suppose that $J_y^j$ does, as shown in Fig. 23a. Then, by the cut property (Lemma 3.8), $\rho \subseteq D(s_\beta ,s_\gamma )$. By transitivity (Lemma 2.4) it also holds that $\rho \subseteq D(s_\beta ,s_\alpha )$. Thus, $e_i$ cannot hit $\rho$. Symmetrically for $J_x^i$. If only $\beta$ passes through $R(\gamma ,\mathcal {P})$, then we can use Lemma 4.10 to derive that $\rho \subseteq D(s_\beta ,s_\gamma )$; the rest follows.
  Suppose that $\rho \subseteq \varGamma$. Then either $\rho$ itself is part of an edge of $J_y^j$ or of $J_x^i$, or $\beta$ passes through $R(\alpha ,\mathcal {P})$ and $\rho$ is at opposite side of it than $\alpha$. In the former case, $\rho \subseteq D(s_\beta ,s_\alpha )$ by the definition of a $\varGamma$-edge in the merge curve. In the latter case, the same is derived by Lemma 3.6 and transitivity (Lemma 2.4). Thus, $e_i$ cannot hit $\rho$.
(iii) Edge $e_i$ (resp. $e_{m-j}$) cannot hit $\partial R_3$, because if it did, $e_i$ and $e_{m-j}$ would not appear sequentially on $R(\alpha _i,\mathcal {P})$ contradicting Lemma 3.8.
(iv) It remains to show that $e_i$ and $e_{m-j}$ cannot both hit $\alpha _i$; however, this is already shown in Lemma 4.11.

Suppose now that (2) holds, see Fig. 23b. Let $R(\gamma ,\mathcal {P})$ be a region in $\mathcal {V}_l(\mathcal {P})$ incident to the $\varGamma$-arc *g* and let $\rho = R(\gamma ,\mathcal {P})\cap g$ be the $\varGamma$-arc bounding $R(\gamma ,\mathcal {P})$, which lies between $v_i$ and $v_{m-j+1}$. At least one of $J_y^j$ or $J_x^i$ or $\beta$ must pass through $R(\gamma ,\mathcal {P})$. By the exact same arguments as before, $\rho \subseteq D(s_\beta ,s_\gamma )$. We infer that there is no bisector $J(s_\beta ,s_\gamma )$ in $R(\gamma ,\mathcal {P})$, for any region $R(\gamma ,\mathcal {P})$ incident to *g* between $v_i$ and $v_{m-j+1}$. Thus, $e_{i+1}=e_{m-j+1}\subseteq g$.

We conclude that in both (1) and (2), $v_{i+1}=v_{m-j+1}$, $v_{m-j}=v_i$, and $m=i+j$. $J(\beta )$ is the concatenation of $J_x^i$ and $J_y^j$ with $e_{i+1}=e_{m-j+1}$. $\square$

#### Lemma 4.14

Suppose vertex $v_{i+1}$ is valid and $e_{i+1}\subseteq J(s_\beta ,s_{a_{i+1}})$. Then $R(\alpha _{i+1},\mathcal {P})$ has not been visited by $J_x^i$ nor $J_y^j$, i.e., $\alpha _{i+1}\ne \alpha _\ell$ for $\ell \le i$ and for $m-j<\ell$.

**Fig. 24:**
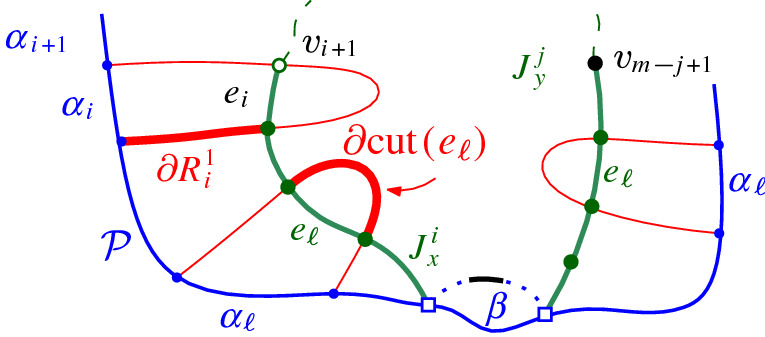
Illustration for Lemma 4.14

#### Proof

Let $e_k, k \le i$, be a bisector edge of $J_x^i$. Denote by $\partial R_k^1$ the portion of $\partial R(\alpha _k,\mathcal {P})$ from $\alpha _k$ to $v_k$ in a counterclockwise traversal, see the bold red part $\partial R_i^1$ in Fig. 24. Analogously, for a bisector edge $e_{m-j}$ of $J_y^j$, where $\partial R_{m-j}^1$ is defined in a clockwise traversal of $\partial R(\alpha _{m-j},\mathcal {P})$. Recall that $cut (e_k)$ denotes the portion of $R(\alpha _k,\mathcal {P})$ *cut out* by edge $e_k$, at opposite side from $\alpha _k$.

The cut property of Lemma 3.8 implies that $v_{i+1}$ cannot be on $\partial cut (e_\ell )$ for any $\ell$, $\ell < i$ and $m-j<\ell$, and that $v_{i+1}$ cannot be on $\partial R_{i}^1$. This implies that $v_{i+1}$ cannot be on $\partial R_{\ell }^1$ for any $\ell < i$, because we have a plane graph in $D_\mathcal {P}$ and by its layout $\partial R_\ell ^1$ is not reachable from $e_{i}$ without first hitting $\partial cut (e_\ell )$ or $\partial R_i^1$. See Fig. 24. Thus, $v_{i+1}$ cannot be on $\partial R(\alpha _\ell )$, $\ell <i$. By Lemma 4.13, $v_{i+1}$ cannot be on $\partial R_{m-j}^1$. This implies, again by the layout, that $v_{i+1}$ cannot be on $\partial R_{\ell }^1$ for all $\ell >m-j$. Thus, $v_{i+1}$ cannot be on $\partial R(\alpha _\ell ,\mathcal {P})$, for any $\ell >m-j$. This implies that $\alpha _{i+1}\ne \alpha _\ell$, for any $\ell$, $\ell \le i$ or $\ell >m-j$. $\square$

By Lemma 4.14, $J_x^{i+1}$ and $J_y^{j+1}$ always enter a new region of $\mathcal {V}_l(\mathcal {P})$ that has not been visited by a lower index edge. Hence, conditions (1) or (2) of Lemma 4.13 must be fulfilled at some point of the induction, completing the proof of Theorem 4.3.

Completing the bi-directional induction establishes also the remaining properties for $J(\beta )$. First, $J(\beta )$ can never enter the same region twice (by Lemma 4.14), except the region of $\alpha _1$, if $\alpha _1=\alpha _m$. The latter is Observation 4.1 (c), where arc $\beta$ splits a single arc $\alpha \in \mathcal {P}$. In this case $J(\beta )$ enters $R(\alpha ,\mathcal {P})$ exactly twice and both $e_1,e_{m-1} \subseteq J(s_\alpha ,s_\beta )$. This is because $J(\beta )$ must intersect $\partial R(\alpha ,\mathcal {P})$, i.e., $J(\beta ) \not \subseteq R(\alpha ,\mathcal {P})$, as otherwise $J(\beta )=J(s_\alpha ,s_\beta )$ (see Fig. 15) contradicting the labeling of the cut property in Lemma 3.8.

Completing the induction for Theorem 4.3 establishes also that $J(\beta )$ is unique and that the conditions of Lemmas 4.11 and 4.12 can never be met. Thus, no vertex of $J(\beta )$, except its endpoints, can be on a boundary arc of $\mathcal {P}$.

## $\mathcal {V}_l(\mathcal {P})$ is Unique

In this section we prove Theorem 3.9 and establish that the Voronoi-like diagram $\mathcal {V}_l(\mathcal {P})$ is unique, for any boundary curve $\mathcal {P}$ on . We first use Theorem 4.3 to show an essential property of Voronoi-like regions, which completes and extends the *cut property* of Lemma 3.8.

**Fig. 25:**
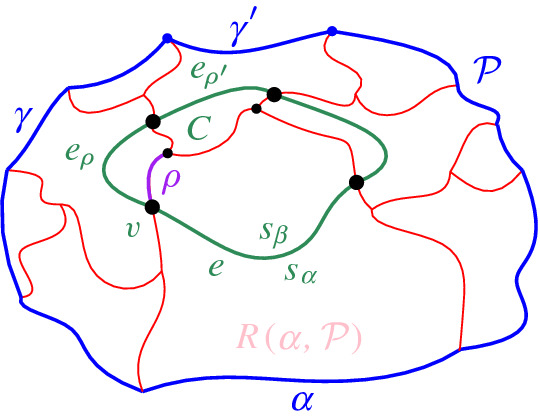
A component *e* of $J(s_\alpha ,\,{\cdot }\,)$ in $R(\alpha ,\mathcal {P})$ as in Lemma 5.1

**Fig. 26:**
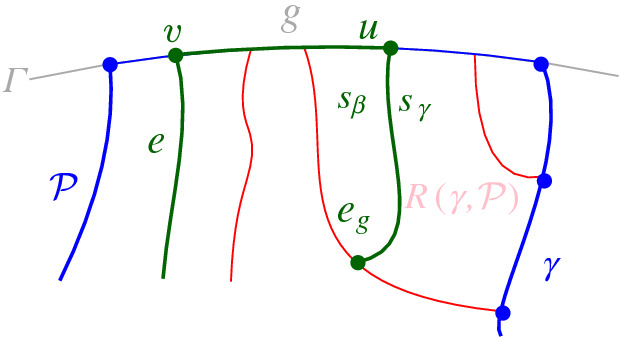
A component *e* of $J(s_\alpha ,\,{\cdot }\,)$ in $R(\alpha ,\mathcal {P})$ with its endpoint *v* on a $\varGamma$-arc *g* as in Lemma 5.1

### Lemma 5.1

Let $\mathcal {P}$ be a boundary curve on , $\mathcal {P}\ne \mathcal {E}$, and let $\alpha ,\beta \in \mathcal {P}$ be two arcs such that $s_\alpha \ne s_\beta$. Suppose that $J(s_\alpha ,s_\beta )$ intersects $R(\alpha ,\mathcal {P})$ with a component *e*, $e\subseteq J(s_\alpha ,s_\beta )\cap R(\alpha ,\mathcal {P})$. Then, $J(s, s_\beta )$ must intersect the domain $D_\mathcal {P}$. Further, there exists a component $\beta '$ of $J(s,s_\beta )\cap D_\mathcal {P}$ such that the merge curve $J(\beta ')$ in $\mathcal {V}_l(\mathcal {P})$ contains *e*, i.e., $e\subseteq \partial R(\beta ',\mathcal {P}\oplus \beta ')$.

We say that the arc $\beta '$ is *missing from* $\mathcal {P}$.

### Proof

Suppose that a component *e* of $J(s_\alpha ,s_\beta )$ intersects $R(\alpha ,\mathcal {P})$, however, $J(s,s_\beta )$ does not intersect $D_\mathcal {P}$, i.e., $D_\mathcal {P}\subseteq D(s,s_\beta )$. Then, for any arc $\chi \in \mathcal {P}$, $\chi \subseteq \mathcal {J}(s,s_\chi )$ and $\chi \subseteq D(s_\chi ,s_\beta )$, by the transitivity of dominance regions (Lemma 2.4). Let $cut (e)$ denote the portion of $R(\alpha ,\mathcal {P})$ cut out by *e*, at opposite side from $\alpha$, as defined in Lemma 3.8; then $cut (e) \subseteq D(s_\beta ,s_\alpha )$, by Lemma 3.8.

Consider an endpoint *v* of *e*. There are two cases:

(i) If *v* is on an edge $\rho$ incident to regions $R(\alpha ,\mathcal {P})$ and $R(\gamma ,\mathcal {P})$, then $J(s_\beta ,s_{\gamma })$ intersects $R(\gamma ,\mathcal {P})$ by an edge $e_{\rho }$, incident to *v*, leaving $\rho$ and $\gamma$ at opposite sides, since $D_\mathcal {P}\subseteq D(s,s_\beta )$, implying that $\gamma \subseteq D(s_\gamma , s_\beta )$, see Fig. 25.
(ii) If *v* is on a $\varGamma$-arc *g*, let $R(\gamma ,\mathcal {P})$ be the first region after *v* (on the side of *e* labeled $s_\beta$) such that $J(s_\beta ,s_{\gamma })$ intersects $g \cap \overline{R(\gamma ,\mathcal {P})}$ at a point *u* (see Fig. 26). Such a region must exist because for all boundary arcs $\chi \in \mathcal {P}$, including the ones incident to *g*, $\chi \subseteq D(s_\chi ,s_\beta )$. Let $e_{g}$ be the component of $J(s_\beta ,s_{\gamma })\cap R(\gamma ,\mathcal {P})$ incident to *u*.

Therefore, given *e* and *v*, we derive an edge $e'$, either $e'=e_\rho$ or $e'=e_{g}$, with the same properties as *e*, in a different region of $\mathcal {V}_l(\mathcal {P})$. This process repeats and there is no way to break it because for any arc $\chi \in \mathcal {P}$, $\chi \subseteq D(s_\chi , s_\beta )$. Thus, we create a closed curve on $\mathcal {V}_l(\mathcal {P})$ consisting of consecutive pieces of $J(s_\beta ,\,{\cdot }\,)$, possibly interleaved with $\varGamma$-arcs, which has the label $s_\beta$ in its interior. No two edges of this curve can intersect in their interior, within a region $R(\chi , \mathcal {P})$, because these edges would be pieces of the same bisector $J(s_\beta ,s_\chi )$, which in turn would not be a simple curve. For exactly the same reason, the curve may not enter $R(\beta ,\mathcal {P})$. Furthermore, no vertex of the curve can repeat under our general position assumption, as no three $s_\beta$-related bisectors can intersect at the same point. Thus, the closed curve must be an $s_\beta$-cycle *C* that is contained in $D_\mathcal {P}$, see Fig. 25, which contradicts Lemma 3.7. Thus, our assumption that $J(s,s_\beta )\cap D_\mathcal {P}=\emptyset$ was false, and hence, $J(s,s_\beta )$ must intersect $\mathcal {P}$.

**Fig. 27:**
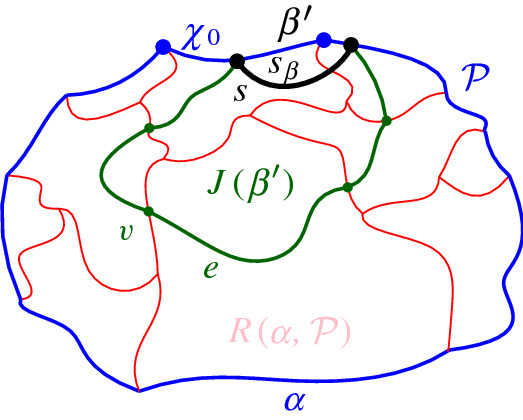
Arc $\beta ' \subseteq J(s,s_\beta )$ in $D_\mathcal {P}$. The merge curve $J(\beta ')$ contains *e*

Let $J_e$ denote the sequence of encountered edges $e_\rho$, starting with the initial edge *e* and ending on the first intersection of an arc $\chi _0$ in $\mathcal {P}$ with $J(s,s_\beta )$. Let $\beta '$ be the component of $J(s,s_\beta )\cap D_\mathcal {P}$ incident to $\chi _0$, see Fig. 27. Clearly $\beta '\ne \beta$, as otherwise $J_e$ would have entered $R(\beta ,\mathcal {P})$. Consider the merge curve $J(\beta ')$ for the arc $\beta '$ on $\mathcal {V}_l(\mathcal {P})$ (see Definition 4.2). By its definition, the path $J_e$ must be a portion of $J(\beta ')$. Since by Theorem 4.3 the merge curve $J(\beta ')$ on $\mathcal {V}_l(\mathcal {P})$ is unique, it follows that $J(\beta ')$ contains $J_e$, and thus, it also contains edge *e*. $\square$

Note that no arc can be *missing* from the envelope  of . We can now prove Theorem 3.9 from Sect. 3.

### Theorem 3.9

Given a boundary curve $\mathcal {P}$ for , $\mathcal {V}_l(\mathcal {P})$ is unique.

### Proof

Let $\mathcal {P}$ be a boundary curve for  such that $\mathcal {P}$ admits a Voronoi-like diagram $\mathcal {V}_l(\mathcal {P})$. Suppose there exist two different Voronoi-like diagrams of $\mathcal {P}$, $\mathcal {V}_l^{(1)} \ne \mathcal {V}_l^{(2)}$. Then there must be an edge $e^{(1)}$ of $\mathcal {V}_l^{(1)}$ bounding regions $R^{(1)}(\alpha ,\mathcal {P})$ and $R^{(1)}(\beta ,\mathcal {P})$ of $\mathcal {V}_l^{(1)}$, where $\alpha ,\beta \in \mathcal {P}$, such that $e^{(1)}$ intersects region $R^{(2)}(\alpha ,\mathcal {P})$ of $\mathcal {V}_l^{(2)}$, since $\alpha$ is common to both $R^{(1)}(\alpha ,\mathcal {P})$ and $R^{(2)}(\alpha ,\mathcal {P})$.

**Fig. 28:**
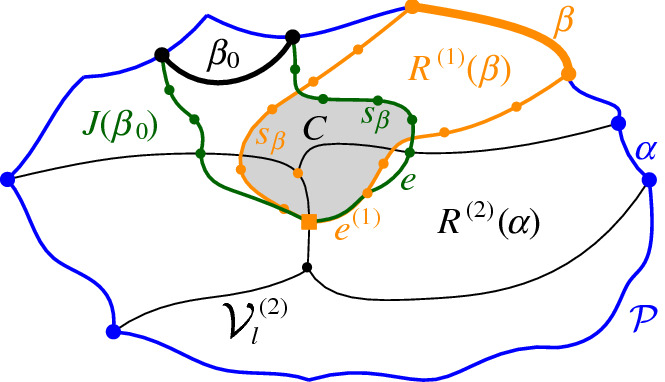
Illustrations for the proof of Theorem 3.9

Let edge $e \subseteq J(s_\beta , s_\alpha )$ be the component of $R^{(2)}(\alpha ,\mathcal {P})\cap J(s_\beta , s_\alpha )$ overlapping with $e^{(1)}$, see Fig. 28. From Lemma 5.1, it follows that there is a non-empty component $\beta _0$ of $J(s,s_\beta )\cap D_\mathcal {P}$ such that $J(\beta _0)$ in $\mathcal {V}_l^{(2)}$ contains edge *e*. Since $J(\beta _0)$ and $\partial R^{(1)}(\beta ,\mathcal {P})$ have an overlapping portion $e\cap e^{(1)}$ and they bound the regions of two different arcs $\beta _0 \ne \beta$ of site $s_\beta$, they form an $s_{\beta }$-cycle *C* as shown in Fig. 28. But *C* is contained in $D_\mathcal {P}$, deriving a contradiction to Lemma 3.7. $\square$

## A Randomized Incremental Algorithm

Consider a random permutation $o =(\alpha _1, \dots , \alpha _h )$ of the set $\mathscr {S}$ of core arcs, where . For $1 \le i \le h$, define the set  to be the subset of the first *i* arcs in *o*, and permutation $o_i=(\alpha _1,\dots , \alpha _i )$. Let $\mathcal {P}_i$ denote the boundary curve derived by the arc insertion operation $\oplus$ by considering arcs in the order $o_i$. Let $D_i$ denote the corresponding domain enclosed by $\mathcal {P}_i$.

Our randomized algorithm is inspired by the randomized, two-phase, approach of Chew [7] for the Voronoi diagram of points in convex position. Here the sites are core arcs in , forming boundary curves, and the algorithm constructs Voronoi-like diagrams within a series of shrinking domains $D_{i}\supseteq D_{i+1}$. The domain of $\mathcal {P}_1$ is $D_1=D(s,s_{\alpha _1})\cap D_\varGamma$; and $D_{h}$ coincides with the Voronoi region ${{\,\textrm{VR}\,}}(s,S)\cap D_\varGamma$. The boundary curves are obtained by the insertion operation $\oplus$, one at each step, starting with $\mathcal {P}_1=J(s,s_{\alpha _1})\cap D_\varGamma$, and ending with $\mathcal {P}_h=\partial {{\,\textrm{VR}\,}}(s,S) \cap D_\varGamma$. The algorithm works in two phases.

In phase 1, the core arcs in  get deleted one by one, in the reverse order of *o*, while recording the neighbors of an arc at the time of its deletion. Let $\mathcal {P}_1 = J(s,s_{\alpha _1})\cap D_\varGamma$, $R(\alpha _1, \mathcal {P}_1) = D(s,s_{\alpha _1})\cap D_\varGamma$, and $\mathcal {V}_l(\mathcal {P}_1)= \emptyset$.

In phase 2, we start with $\mathcal {V}_l(\mathcal {P}_1)$ and incrementally compute $\mathcal {V}_l(\mathcal {P}_{i})$, $i =2, \dots , h$, by inserting arc $\alpha _{i}$ to $\mathcal {P}_{i-1}$, and obtaining $\mathcal {P}_i=\mathcal {P}_{i-1} \oplus \alpha _{i}$, and $\mathcal {V}_l(\mathcal {P}_{i})=\mathcal {V}_l(\mathcal {P}_{i-1})\oplus \alpha _{i}$. When considering an arc $\alpha _i$, we use the information of its recorded neighbors from phase 1 to determine its insertion point. At the end, we obtain $\mathcal {V}_l(\mathcal {P}_h)$, where $\mathcal {P}_h$ is a boundary curve for . Since  has only one boundary curve, it follows that $\mathcal {P}_h$ coincides with $\partial {{\,\textrm{VR}\,}}(s,S) \cap D_\varGamma$.

We have already established the correctness of the insertion operation $\oplus$, thus, the algorithm correctly computes $\mathcal {V}_l(\mathcal {P}_h)$. We have also established that $\mathcal {V}_l(\mathcal {P}_h)$ coincides with the true Voronoi diagram , by Corollary 3.5. Thus, the algorithm correctly computes .

Next we analyze the time complexity of this algorithm and prove that the time complexity of step-*i* is expected *O*(1). Thus, the overall time complexity is expected *O*(*h*).

### Lemma 6.1

$\mathcal {P}_i$ contains at most $i-1$ auxiliary arcs; thus, $|\mathcal {V}_l(\mathcal {P}_i)|=O(i)$.

### Proof

By definition, $|\mathcal {P}_1| =1$. At each step of phase 2, exactly one original arc is inserted, and at most one additional auxiliary arc is created by a split in case (c) of Observation 4.1, except from $i=1$ and $i=h$. Thus, the total number of auxiliary arcs is at most $i-1$ and the number of original arcs is at most *i*. Since an original arc may be merged with its neighbor in case (f) of Observation 4.1, the number of original arcs in $\mathcal {P}_i$ may indeed be less than *i*. Since the complexity of $\mathcal {V}_l(\mathcal {P}_i)$ is $O(|\mathcal {P}_i|)$, the claim follows. $\square$

### Time Analysis of the Randomized Incremental Algorithm, a Variant of Backwards Analysis

**Fig. 29:**
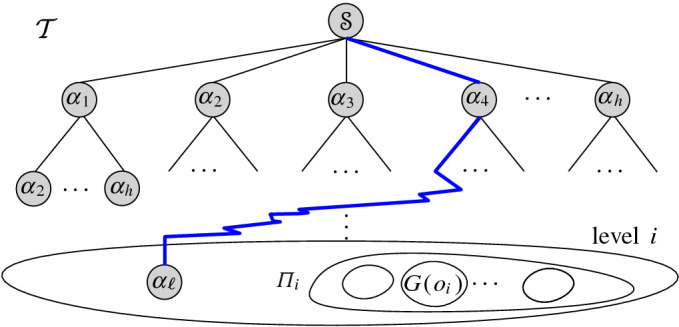
There are $h!/(h-i)!$ nodes nodes at level-*i* of the decision tree $\mathcal {T}$, each corresponding to a unique permutation of *i* core arcs; the label of a node indicates the last element in the permutation. Level *i* is partitioned into disjoint groups of *i* nodes (permutations) each; $(i-1)!$ such groups constitute a block $\varPi _i$. The illustration is schematic, the grouped nodes are not consecutive.

The time complexity of the algorithm, for each step *i*, has been expressed in Lemma 4.7 as a function of the resulting diagram $\mathcal {V}_l(\mathcal {P}_{i})$. This calls for *backwards analysis* to estimate its expectation, see [20]. However, although $\mathcal {V}_l(\mathcal {P}_{i})$ is unique, the boundary curve $\mathcal {P}_i$, and consequently its diagram, depend on the permutation order. As a result, backwards analysis is not directly applicable, contrary to our preliminary paper. In this section we revisit the analysis of [9], and introduce a variation of backwards analysis that is applicable to order-dependent structures.

Consider the *decision tree* $\mathcal {T}$ of all possible random choices that can be made by our incremental algorithm on the input set of core arcs , , see Fig. 29. $\mathcal {T}$ has *h*! leaves each corresponding to one permutation of the arcs in . At level-*i*, there are $h!/(h-i)!$ nodes, and each node corresponds to a unique permutation of *i* core arcs. A set of *i* core arcs  is associated with *i*! different nodes at level-*i*, which are called the *block of* . We have ${h \atopwithdelims ()i}$ distinct such blocks at level-*i*. Although all nodes within one block are associated with the same set of core arcs, their corresponding boundary curves may vary considerably depending on their permutation order.

We use the following strategy. We partition each block at level-*i* into $(i-1)!$ disjoint groups of *i* nodes each. For each group we show that step *i* requires total time *O*(*i*), considering all the *i* permutations within the group. Thus, on average, the algorithm spends *O*(1) time on each node of $\mathcal {T}$. Since all permutations are equally likely, we obtain the expected linear *O*(*h*) time complexity of our algorithm.

Let $o_i= (\alpha _1, \alpha _2, \dots , \alpha _i)$ be an arbitrary permutation of . From $o_i$ we define a group $G = G(o_i)$ of *i* permutations as follows: for each $1 \le j < i$, remove ${\alpha _j}$ from its position in $o_i$ and append it to the end of $o_i$.12

**Fig. 30:**
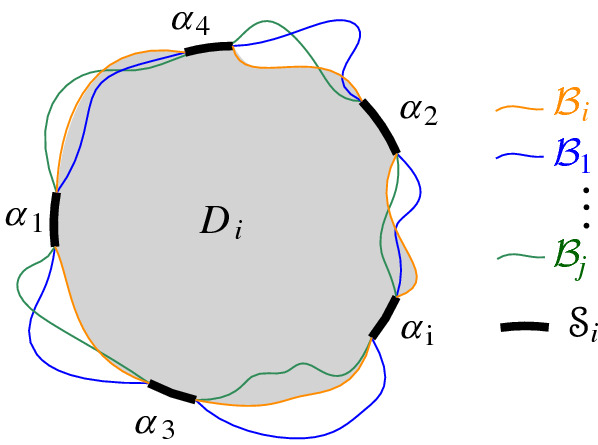
Schematic differences between the boundary curves $\mathcal {B}_1,\dots ,\mathcal {B}_i$. The domain $D_i$ is shown shaded

Let $\mathcal {B}_j$ and $\mathcal {V}_l(\mathcal {B}_j)$, $1\le j\le i$, denote the boundary curves, and their Voronoi-like diagrams, derived incrementally, by arc insertion, following the order $o_j$, see Fig. 30. The boundary curve $\mathcal {B}_i$ is the base one derived by following the order $o_i$ and its domain is denoted $D_i$. In the following we establish relations between $\mathcal {V}_l(\mathcal {B}_j)$ and $\mathcal {V}_l(\mathcal {B}_i)$ so that we can bound the time complexity of step *i* on the entire group $G(o_i)$ (Lemma 6.11).

Before proceeding, we show that it is indeed possible to partition the block $\varPi _i$ of all the *i*! permutations of set  in disjoint groups of *i* permutations each, using the scheme of (1)–(2). The proof of the following lemma was pointed out to us by Stefan Felsner in personal communication (Dec. 2019).

#### Lemma 6.2

The partitioning of $\varPi _i$ into disjoint groups by the scheme we defined in (1)–(2) is possible, i.e., for all $i \in {\mathbb {N}}$ and any block $\varPi _i$ of permutations on  there exists a set $F \subset \varPi _i$ of $(i-1)!$ permutations such that $\varPi _i = \dot{\bigcup }_{o\in F} G(o)$; that is, $G(\pi )\cap G(\sigma )=\emptyset$, for any $\pi , \sigma \in F$.

#### Proof

Following [15], denote by $\left\lfloor \pi \right\rfloor$ the set of all permutations that are obtained from a permutation $\pi$ by deleting one element. Let $F\subseteq \varPi _i$ be a set of permutations such that $\left\lfloor \pi \right\rfloor$ and $\left\lfloor \sigma \right\rfloor$ are disjoint, for each $\pi , \sigma \in F$. Levenshtein calls such a family *F* of $(i-1)!$ permutations a *code capable of correcting single deletions*, and proves that these codes exist for all $i\in {\mathbb {N}}$ [15, Thm. 3.1]. The set $\left\lfloor \pi \right\rfloor$ is equivalent to $G(\pi )$. Since the set *F* exists, it follows that $\varPi _i$ is the disjoint union $\dot{\bigcup }_{o\in F}G(o)$. $\square$

We can now proceed to estimate the time complexity of step *i* on one group of permutations $G(o_i)$. We first introduce some terminology.

#### Definition 6.3

**Fig. 31:**
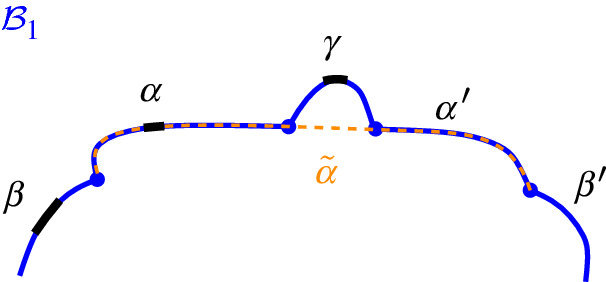
Illustration for Definition 6.3, $o_1 = (\beta , \alpha ,\gamma )$: The core arc  is the source of $\alpha ' \in in _1^+$. The expanded arc $\tilde{\alpha }\supseteq \alpha '$ was created at the time of inserting $\alpha$, while constructing $\mathcal {B}_1$

Let $\alpha '$ be an auxiliary arc in $\mathcal {B}_j$ and let  be a core arc of the same site. We say that $\alpha '$ *is an auxiliary arc of* $\alpha$ if, at step *k*, when $\alpha =\alpha _k$ is inserted in $B_j$, the created original arc $\tilde{\alpha }\supseteq \alpha \cup \alpha '$ (see Fig. 31). The core arc  is called the *source of* $\alpha '$, denoted $source _j(\alpha ')$. If $\alpha '$ appears on $J(s,s_\alpha )$ counterclockwise (resp. clockwise) from its source $\alpha$, then $\alpha '$ is called a *ccw* (resp. *cw*) auxiliary arc. For example in Fig. 31, $\alpha '$ is a cw auxiliary arc of $\alpha$.

The source indicates the core arc in  that creates $\alpha '$.  may contain several core arcs of the same site, but only one of them is the source of $\alpha '$.

**Fig. 32:**
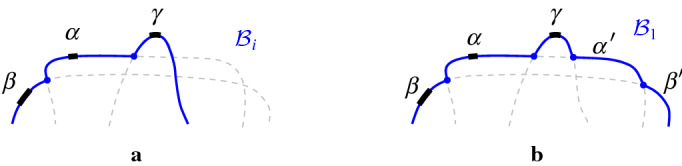
**a** Boundary curve $\mathcal {B}_i$, where $o_i= (\gamma , \beta , \alpha )$. **b** Boundary curve $\mathcal {B}_1$, where $o_1= (\beta , \alpha , \gamma )$, containing arcs $\alpha ', \beta ' \in in _1$, because ${\gamma }$ was inserted last

The boundary curves $\mathcal {B}_j$, $j<i$, may get in and out of the domain $D_i$, see Fig. 30. To identify their differences from $\mathcal {B}_i$, let $in _j = \mathcal {B}_j \cap D_{i}$, and $out _j = \mathcal {B}_j \setminus \overline{D_i}$, denote the portion of $\mathcal {B}_j$ inside, and outside of $D_i$, respectively. We partition the auxiliary arcs of $in _j$ into $in _j^+$ and $in _j^-$, where $in _j^+$ (resp. $in _j^-$) includes the ccw (resp. cw) auxiliary arcs of $in _j$, see Fig. 32. In the following we only consider $in _j^+$ as $in _j^-$ is symmetric.

#### Observation 6.4

The boundary curve $\mathcal {B}_j$, $j\ne i$, contains no auxiliary arcs of $\alpha _j$, as $\alpha _j$ appears last in $o_j$. All arcs in $\mathcal {B}_i$ appear in $\mathcal {B}_j$ except any auxiliary arcs of $\alpha _j$. No arc of $out _j$ can have a region adjacent to $R(\alpha _j,\mathcal {B}_j)$ in $\mathcal {V}_l(\mathcal {B}_j)$.

#### Proof

Since the insertion order of all core arcs, except $\alpha _j$, is identical in $o_i$ and $o_j$, it follows that all auxiliary arcs of $\mathcal {B}_i$, except any auxiliary arcs of $\alpha _j$, must also appear in $\mathcal {B}_j$.

**Fig. 33:**
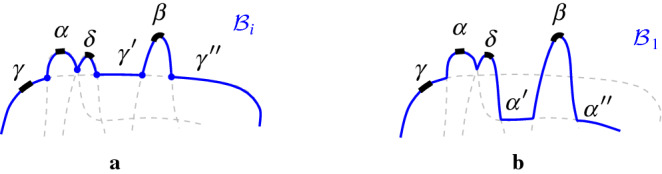
**a** Boundary curve $\mathcal {B}_i$, where $o_i= (\gamma , \alpha , \beta , \delta )$. **b** Boundary curve $\mathcal {B}_1$ containing arcs $\alpha ',\alpha ''$ in $out _1$, where $o_1 = (\alpha , \beta , \delta , \gamma )$

Observe that any auxiliary arc $\alpha '\in out _j$ must lie below (as seen from $D_i$) an auxiliary arc in $\mathcal {B}_i$, by the definition of $out_j$, and the fact that $\mathcal {B}_i$ and $\mathcal {B}_j$ are defined on the same set of core arcs. Thus, $\alpha '$ must lie below an auxiliary arc of $\alpha _j$, see Fig. 33, where $\alpha '$ and $\alpha ''$ in $\mathcal {B}_1$ lie below the auxiliary arcs $\gamma '$ and $\gamma ''$ of $\alpha _1=\gamma$ in $\mathcal {B}_i$. Since arcs of the same site cannot have adjacent regions, no auxiliary arc of $\alpha _j$ can have a region adjacent to $R(\alpha _j,\mathcal {B}_j)$; the claim follows. $\square$

#### Observation 6.5

Let $\alpha ' \in in _j$ and let $\alpha _k = source _j(\alpha ')$. Then $k>j$, i.e., $\alpha _k$ follows $\alpha _j$ in $o_i$. Further, if $\alpha ' \in in _j^+$ then $(\alpha _k,\alpha _j,\alpha ')$ appear ccw in $\mathcal {B}_j$.

#### Observation 6.6

**Fig. 34:**
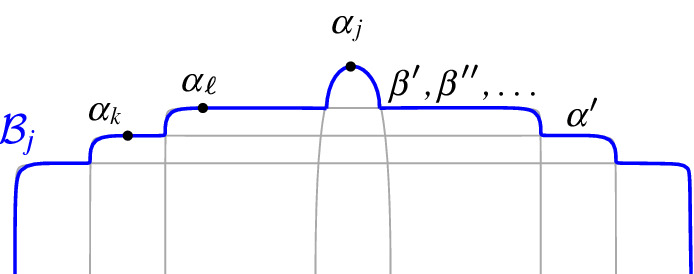
If $\alpha ',\beta ' \in in _j^+$, then $j<k< \ell$ and $(\alpha _k, \alpha _\ell , \alpha _j,\beta ', \alpha ')$ appear in ccw order on $\mathcal {B}_j$

Figure 34 indicates the structure of $in _j^+$. Let $\alpha ',\beta '\in in _j^+$ such that $\alpha _k =source _j(\alpha ')$, $\alpha _\ell =source _j(\beta ')$, and $k<\ell$. Then $j<k< \ell$ and $(\alpha _k, \alpha _\ell , \alpha _j, \beta ',\alpha ')$ appear in ccw order along $\mathcal {B}_j$. Further, all auxiliary arcs of $\alpha _\ell$ must appear before the auxiliary arcs of $\alpha _k$ as we move on $\mathcal {B}_j$ counterclockwise from $\alpha _j$.

Since many auxiliary arcs of $$in _j^+$$ can have the same source, we defineAll arcs in $$N_j$$ are of different sites. Sets $$in ^+_j$$ and $$in ^+_k $$, $$k \ne j$$, may contain many common arcs, however, we have the following disjointness property.

#### Lemma 6.7

$N_j \cap N_k = \emptyset$, for all $k \ne j$. Thus, $\sum _{j =1}^i |N_j| = O(i)$.

#### Proof

Suppose $\alpha _\ell \in N_j \cap N_k$ and $j<k$, then $\alpha _\ell = source _j(\alpha ')$, where $\alpha ' \in in ^+_j$ and $\alpha _\ell =source _k(\alpha '')$, where $\alpha '' \in in ^+_k$. (The arcs $\alpha '$ and $\alpha ''$ may or may not overlap). By Observation 6.5, $j<\ell$ (resp. $k<\ell$) and $(\alpha _\ell ,\alpha _j, \alpha ')$ (resp. $(\alpha _\ell ,\alpha _k, \alpha '')$) must appear in ccw order on $\mathcal {B}_j$ (resp. $\mathcal {B}_k$).

**Fig. 35:**
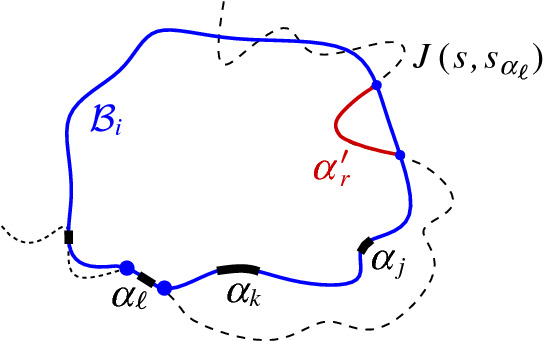
Illustration for Lemma 6.7. The case $(\alpha _\ell ,\alpha _k,\alpha _j)$ appear ccw

**Fig. 36:**
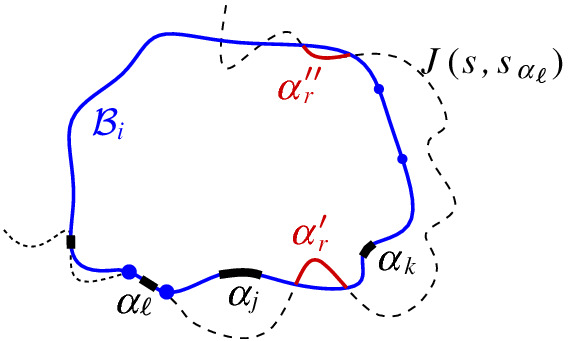
Illustration for Lemma 6.7. The case $(\alpha _\ell ,\alpha _j,\alpha _k)$ appear ccw

Suppose first that $(\alpha _\ell ,\alpha _k,\alpha _j)$ appear in ccw order on $\mathcal {B}_i$. Then, since $k<\ell$, the arc $\alpha _k$ is inserted before $\alpha _\ell$ in $\mathcal {B}_j$, and thus, $\alpha '$ cannot exist in $\mathcal {B}_j$, see Fig. 35. Suppose now that $(\alpha _\ell ,\alpha _j,\alpha _k)$ appear in ccw order on $\mathcal {B}_i$. Then, since $j<\ell$, the arc $\alpha _j$ is inserted before $\alpha _\ell$ in $\mathcal {B}_k$, thus, $\alpha ''$ cannot exist on $\mathcal {B}_k$, see Fig. 36. In either case we derive a contradiction. $\square$

Next we establish that the parameters of the time complexity analysis for step *i*, as given in Definition 4.6 and Lemma 4.7, sum up to *O*(*i*) on all boundary curves $\mathcal {B}_j$, $j\le i$.

#### Lemma 6.8

Considering all the boundary curves of group $G(o_i)$,

$$\begin{aligned} \sum _{j=1}^i\bigl (d_1(\alpha _j,\mathcal {B}_j) +d_2(\alpha _j,\mathcal {B}_j) +{\tilde{d}}(\alpha _j,\mathcal {B}_j)\bigr )= O(i). \end{aligned}$$

#### Proof

Let $\alpha$ and $\gamma$ denote the original arcs preceding and following $\alpha _j$ respectively in $\mathcal {B}_i$ (equiv. in $\mathcal {B}_j$). Let $d(\alpha _j,\mathcal {B}_k)$ denote the auxiliary arcs on the boundary curve $\mathcal {B}_k$, $k=i,j$, from $\alpha$ to $\gamma$.

We first observe that $$d(\alpha _j,\mathcal {B}_j)$$ cannot contain any portion of $$out _j$$ because no auxiliary arc of $$\alpha _j$$ may appear in $$\mathcal {B}_i$$ from $$\alpha $$ to $$\gamma $$, since $$\alpha _j$$ is the only core arc on $$\mathcal {B}_i$$ between $$\alpha $$ to $$\gamma $$. Thus, we only need to consider the auxiliary arcs of $$in _j$$. Next, we observe that no two auxiliary arcs in $$d(\alpha _j,\mathcal {B}_j)$$ can have the same source in $$ N_j$$ for the same reason, i.e., there is no core arc from $$\alpha $$ to $$\gamma $$ except $$\alpha _j$$. Thus, we can bound $$d(\alpha _j,\mathcal {B}_j)\le d(\alpha _j,\mathcal {B}_i) + |N_j|$$. Then, by Lemma 6.7,

$$\begin{aligned} \sum _{j=1}^id(\alpha _j,\mathcal {B}_j) \le |\mathcal {B}_i| + O(i) = O(i). \end{aligned}$$

Since $d_1(\alpha _j,\mathcal {B}_j) + d_2(\alpha _j,\mathcal {B}_j) \le d(\alpha _j,\mathcal {B}_j)$, it follows

$$\begin{aligned} \sum _{j=1}^i\,(d_1(\alpha _j,\mathcal {B}_j) + d_2(\alpha _j,\mathcal {B}_j)) = O(i). \end{aligned}$$

If ${\tilde{d}}(\alpha _j,\mathcal {B}_j)>0$, we have case (d) of Observation 4.1. In this case, the endpoints of $\alpha _j$ are incident to $\varGamma$, both in $\mathcal {B}_j$ and $\mathcal {B}_i$. Then, by Observations 6.4 and 6.6, we have both $in _j = \emptyset$ and $out _j = \emptyset$, implying that $\mathcal {B}_j=\mathcal {B}_i$; thus, ${\tilde{d}}(\alpha _j,\mathcal {B}_j)= {\tilde{d}}(\alpha _j,\mathcal {B}_i)$. Then,

$$\begin{aligned} \sum _{j=1}^i|{\tilde{d}}({\alpha _j},\mathcal {B}_j)| \le |{\tilde{\mathcal {B}}}_i| = O(i). \end{aligned}$$

$$\square $$

**Fig. 37:**
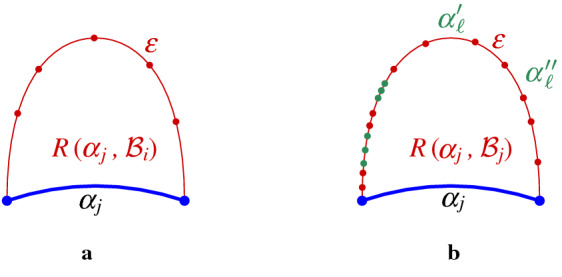
Illustration for Lemma 6.9. Between any two consecutive adjacencies of $R(\alpha _j,\mathcal {B}_j)$ with regions of auxiliary arcs in $in _j$ of the same source, there must be an adjacency with an arc $\varepsilon \in \mathcal {B}_j\cap \mathcal {B}_i$

#### Lemma 6.9

$$|R(\alpha _j,\mathcal {B}_j)| \le 2|R({\alpha _j},\mathcal {B}_i)| + |N_j|$$.

#### Proof

We compare $R(\alpha _j,\mathcal {B}_j)$ and $R({\alpha _j},\mathcal {B}_i)$ and bound differences in their adjacencies. By Observation 6.4 no arc in $out _j$ can have a region adjacent to $R(\alpha _j,\mathcal {B}_j)$. We also observe the following: if an arc $\varepsilon \in \mathcal {B}_j\cap \mathcal {B}_i$, common to both $\mathcal {B}_j$ and $\mathcal {B}_i$, has a region $R(\varepsilon ,\mathcal {B}_j)$ adjacent to $R(\alpha _j,\mathcal {B}_j)$ in $\mathcal {V}_l(\mathcal {B}_j)$, then $R(\varepsilon ,\mathcal {B}_i)$ must also be adjacent to $R({\alpha _j},\mathcal {B}_i)$ in $\mathcal {V}_l(\mathcal {B}_i)$, see Fig. 37. This is correct, because otherwise, the Voronoi edge *e* bounding $R(\alpha _j,\mathcal {B}_j)$ and $R(\varepsilon ,\mathcal {B}_j)$ (or a portion of it) would be contained in a region $R(\eta , B_i)$ for an arc $\eta$ that does not appear in $\mathcal {B}_j$, i.e., $\eta \in out _j$. By Observation 6.4, this arc may only be an auxiliary arc of $\alpha _j$. However, by Lemma 5.1, if we insert $\eta$ to $\mathcal {V}_l(B_j)$, the region $R(\eta ,B_j\oplus \eta )$ will contain a portion of the edge *e*, thus, it will be adjacent to $R(\alpha _j,B_j\oplus \eta )$, deriving a contradiction, as arcs of the same site cannot have adjacent regions.

Let $|R(\alpha _j,\mathcal {B}_j)|_x$ denote the number of additional adjacencies that $R(\alpha _j,\mathcal {B}_j)$ may have over $R(\alpha _j,\mathcal {B}_i)$, i.e., $|R(\alpha _j,\mathcal {B}_j)| \le |R(\alpha _j,\mathcal {B}_i)| + |R(\alpha _j,\mathcal {B}_j)|_x$. We show that $|R(\alpha _j,\mathcal {B}_j)|_x\le |R(\alpha _j,\mathcal {B}_i)| +|N_j|$. Since auxiliary arcs of the same site can never have adjacent regions, it follows that between any two possible new adjacencies of $R(\alpha _j,\mathcal {B}_j)$ with auxiliary arcs of the same source in $in _j$, there must be an adjacency with some arc that is common to both $\mathcal {B}_i$ and $\mathcal {B}_j$.

Since by Observation 6.6 auxiliary arcs of one source in $N_j$ must appear in a certain order along $\mathcal {B}_j$, and they cannot alternate, the bound follows. $\square$

#### Lemma 6.10

Consider case (c) of Observation 4.1 at the insertion of $\alpha _j$ in $\mathcal {B}_j$. Suppose that the insertion of $\alpha _j$ splits an existing arc $\omega$ into two pieces $\omega _1$ and $\omega _2$. Then at least one of these two arcs (say $\omega _1$) must also exist in $\mathcal {B}_i$. Further, $|R({\omega _1},\mathcal {B}_j)| \le 2|R({\omega _1},\mathcal {B}_i)| + |N_j|$.

#### Proof

**Fig. 38:**
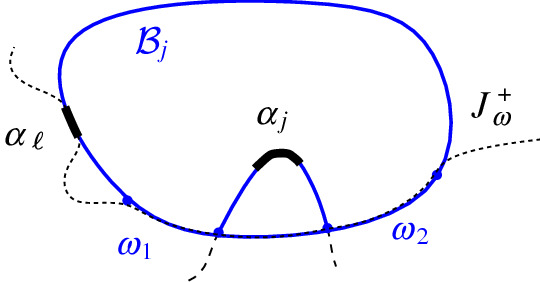
Illustration for the proof of Lemma 6.10. If $\omega _2\notin \mathcal {B}_i$, then $\omega _1\in \mathcal {B}_i$

Suppose $\omega _1\alpha _j\omega _2$ appear in $\mathcal {B}_j$ in ccw order and $\omega _2\notin \mathcal {B}_i$. Then $\omega _2 \in in _j^+$, see Fig. 38. Let $\alpha _\ell =source _j(\omega _2)$, then $\ell >j$ as $\omega _2 \in in _j^+$. We claim that $\omega _1$ must belong to $\mathcal {B}_i$.

Let $\tilde{\omega }\supset \alpha _\ell$ denote the expanded arc created at the insertion time of $\alpha _\ell$ following the order $o_j$. Clearly, $\tilde{\omega }\supset \omega$. Let $\hat{\omega }\supset \alpha _\ell$ denote the expanded arc created at the insertion time of $\alpha _\ell$, following $o_i$. Since $\ell >j$, it follows that $\hat{\omega }$ can extend ccw at most until $\alpha _j$ and $\hat{\omega }\subset \tilde{\omega }$. Since $\tilde{\omega }$ extends ccw past $\alpha _j$, it follows that no core arc $\alpha _\rho$, with $\rho <\ell$ can exist between $\alpha _l$ and $\alpha _j$. Thus, $\hat{\omega }$ must extend ccw to $\alpha _j$ and $\hat{\omega }\supset \omega _1$. In addition, no $\alpha _\rho$, with $\rho >\ell$, can delete $\omega _1$ during its insertion, while following $o_i$, because the same would happen in $o_j$ and $\omega _1$ exists in $\mathcal {B}_j$. Thus, $\omega _1$ must exist in $\mathcal {B}_i$.

We can now bound $|R({\omega _1},\mathcal {B}_j)| \le 2|R({\omega _1},\mathcal {B}_i)| + |N_j|$ analogously to Lemma 6.9. The only additional argument needed for the fact that no arc in $out _j$ can have a region adjacent to $R(\omega _1,\mathcal {B}_j)$ is the observation that each arc in $out _j$ lies below the $s_{\omega }$-bisector, because arc $\alpha _j$ splits arc $\omega$ (case (c) of Observation 4.1). $\square$

Let $T(i,o_j)$ denote the time complexity of step-*i* following permutation $o_j$, i.e., the time required by the last arc insertion of $o_j$.

#### Lemma 6.11

The time for step-*i* on the entire group $G=G(o_i)$ is

$$\begin{aligned} T(i,G)= \sum _{o_j \in G} T(i,o_j) =O(i). \end{aligned}$$

#### Proof

Lemmas 6.9 and 6.10 establish that $|R(\alpha _j,\mathcal {B}_j)| + |R(\omega _j,\mathcal {B}_j)|\le 2(|R({\alpha _j},\mathcal {B}_i)| +|R(\omega _j,\mathcal {B}_i)| + |N_j|)$, where $\omega _j$ denotes one of the two arcs that is split and belongs to $\mathcal {B}_i$, if case (c) of Observation 4.1 is concerned. Since $\omega _j$ is always an immediate neighbor of $\alpha _j$, we count it at most twice, and thus, the total complexity $\sum _{j=1}^i |R(\omega _j,\mathcal {B}_i)|$ is *O*(*i*). Together with Lemma 6.7 this directly implies that $\sum _{j=1}^i(|R(\alpha _j,\mathcal {B}_j)| + r(\alpha _j,\mathcal {B}_j)) = O(i)$. Lemma 6.8 establishes that

$$\begin{aligned} \sum _{j=1}^i\,\bigl (d_1(\alpha _j,\mathcal {B}_j) +d_2(\alpha _j,\mathcal {B}_j) +{\tilde{d}}(\alpha _j,\mathcal {B}_j)\bigr ) = O(i). \end{aligned}$$

Then by Lemma 4.7 the claim is derived. $\square$

All permutations at level-*i* of the decision tree are equally likely. By Lemma 6.2, it is possible to partition them into groups of *i* nodes each, which satisfy our scheme of (1)–(2). By Lemma 6.11, each group requires total *O*(*i*) time to perform step *i* on all its permutations. We thus conclude:

#### Theorem 6.12

The time complexity of step *i* of the randomized algorithm is expected *O*(1).

We conclude with the following theorem.

#### Theorem 6.13

Given an abstract Voronoi diagram $\mathcal {V}(S)$, the diagram $\mathcal {V}(S\setminus \{s\})\cap {{\,\textrm{VR}\,}}(s,S)$ can be computed in expected *O*(*h*) time, where *h* is the complexity of $\partial {{\,\textrm{VR}\,}}(s,S)$. Thus, the updated Voronoi diagram $\mathcal {V}(S\setminus \{s\})$ can be computed from $\mathcal {V}(S)$, after the deletion of site *s*, in expected linear time *O*(*h*).

## Computing the Order-*k* Voronoi Diagram Iteratively

Our algorithm to perform deletion in expected linear-time can be adapted to iteratively compute the order-*k* abstract Voronoi diagram, for increasing values of *k*, in total time $O(k(n-k) n + n\log n)$, if $k\le n/2$. In particular, given a face $f$ of an order-*k* Voronoi region, we can compute the order-$(k\,{+}\,1)$ subdivision within $f$ in expected time $O(|\partial f|)$. In this section we describe the required adaptation over site-deletion.

The *order*-*k* *abstract Voronoi region* of a subset of sites $H \subset S$, $|H|=k$, is defined [3] as

$$\begin{aligned} \text{ VR}_k{(}H, S)\,=\!\bigcap _{\begin{array}{c} q \in H\\ p \in S \setminus H \end{array}}\!D(q,p). \end{aligned}$$

The *order*-*k* *abstract Voronoi diagram* of *S* is [3]

$$\begin{aligned} \mathcal {V}_k(S) \,=\, {\mathbb {R}}^2\setminus \!\bigcup _{\begin{array}{c} H \subset S\\ |H| = k \end{array}}\!\text{ VR}_k{(}H, S). \end{aligned}$$

The combinatorial complexity of $\mathcal {V}_k(S)$ is $O(k(n-k))$. For $k=1$, it is the nearest-neighbor abstract Voronoi diagram $\mathcal {V}(S)$, and for $k=n-1$, it is the farthest abstract Voronoi diagram $\textrm{FVD}(S)$. The vertices of the diagram are classified into *new* and *old*, where a *new* vertex in $\mathcal {V}_k(S)$ is an *old* vertex of $\mathcal {V}_{k+1}(S)$.

Consider a face $f$ of an order-*k* Voronoi region $\text{ VR}_k{(}H)$, $H\subset S$, $|H|=k$. Let $S_f\subseteq S\setminus H$ denote the set of sites, which together with *H*, induce the Voronoi edges on the boundary $\partial f$. Our goal is to compute the Voronoi diagram of $S\setminus H$ within $f$, $\mathcal {V}(S_f) \cap f$, in expected linear time, i.e., in time $O(|\partial f|)$. This diagram is a tree (or forest if $f$ is unbounded) with properties analogous to Lemma 2.1 (see also [5]). To extend Theorem 6.13 from $k=1$ to an arbitrary *k*, there is a non-trivial challenge to overcome: the complexity of the boundary $\partial f$ depends not only on $|S_f|$ but also on *k*. Thus, a direct application of our deletion algorithm would not result in a linear-time scheme, if *k* is not a constant.

Consider a face $f$ of $\text{ VR}_k{(}H, S)$ and its boundary $\partial f$. We call any piece of $\partial f$ between two consecutive *new* vertices, an *order*-*k* *arc*. Such an arc does not have constant complexity but may contain a sequence of old Voronoi vertices on $\partial f$. In this section, let  denote the collection of the order-*k* arcs along the boundary of $f$.

An order-*k* arc $\alpha$, bounding the face *f*, is a piece of the so-called *Hausdorff bisector* between site $s_\alpha \in S_f$ and set *H* (see, e.g., [18] for the definition of the concrete Hausdorff bisector between two point sets). In abstract terms, the *Hausdorff bisector* between $s_\alpha$ and *H* is the boundary of the farthest Voronoi region $\text{ FVR }(s_\alpha , H \cup \{s_\alpha \})$, where $\text{ FVR }(s,S') = \bigcap _{q \in S' \setminus \{s\}} D(q,s)$.

Let the *Hausdorff bisector* between a site $s_\alpha \in S_f$ and *H*, which is relevant to face *f*, be defined as

$$\begin{aligned} J(s_\alpha , H) = \partial \text{ FVR }(\alpha , H \cup \{s_\alpha \}), \end{aligned}$$

where $\text{ FVR }(\alpha , H \cup \{s_\alpha \})$ denotes the face of region $\text{ FVR }(s_\alpha , H \cup \{s_\alpha \})$ that is incident to arc $\alpha$. $J(s_\alpha ,H)$ is an unbounded Jordan curve dividing the plane in two open parts; let $D(s_\alpha ,H)=\text{ FVR }(\alpha , H \cup \{s_\alpha \})$.

The complexity of $J(s_\alpha , H)$ is $\varTheta (|H|)$, and this is an obstacle to our randomized linear time scheme. It is possible to overcome this problem by considering relaxed Hausdorff bisectors whose complexity depends solely on order-*k* arcs, and which define a series of even larger shrinking domains enclosing the face $f$. Let $H_\alpha \subseteq H$ be the subset of sites in *H* that, together with $s_\alpha$, define the edges and vertices along the arc $\alpha$. Instead of $J(s_\alpha ,H)$, which is hard to compute, we consider the Hausdorff bisector $J(s_\alpha ,H_\alpha )$, where $\alpha \subseteq J(s_\alpha ,H_\alpha )$, and has complexity $\varTheta (|H_\alpha |)$. In fact, $\alpha \subseteq J(s_\alpha , {\tilde{H}}_\alpha )$, for any $H_\alpha \subseteq {\tilde{H}}_\alpha \subseteq H$. Let $|\alpha |$ denote the complexity of arc $\alpha$, $|\alpha |= |H_\alpha |$. We make use of the following property.

### Lemma 7.1

$$J(s_\alpha ,H)\subseteq \overline{D(s_\alpha ,{\tilde{H}}_\alpha )}\subseteq \overline{D(s_\alpha ,H_\alpha )}$$, where $$H_\alpha \subseteq {\tilde{H}}_\alpha \subseteq H$$.

### Proof

Since $H_\alpha \subseteq H$, we have

3 $$\begin{aligned} D(s_\alpha ,H) = \text{ FVR }(s_\alpha , H \cup \{s_\alpha \})\subseteq \text{ FVR }(s_\alpha , H_\alpha \cup \{s_\alpha \})= D(s_\alpha ,H_\alpha ). \end{aligned}$$

Thus, it holds $J(s_\alpha ,H) = \partial {D(s_\alpha ,H)} \subseteq \overline{D(s_\alpha ,H_\alpha )}$. Analogously we can show the subset relation for ${\tilde{H}}_\alpha$. $\square$

It is now straightforward to adapt the algorithm of Sect. 6, using appropriate Hausdorff bisectors that are derived by the order-*k* arcs in , in place of the *s*-related bisectors in the previous sections. The complexity of each such Hausdorff bisector must be proportional to the complexity of its underlying order-*k* arc. Lemma 7.1 implies the correctness of this adaptation.

We start with domain $D_1$ defined by $J(s_{\alpha _1}, H_{\alpha _1})$, i.e., $D_1=D(s_{\alpha _1}, H_{\alpha _1})\cap D_\varGamma$, for the first order-*k* arc $\alpha _1$ of a random permutation of . The boundary complexity of $D_1$ is $O(|\alpha _1|)$.

Note that $D_1$ is a superset of domain $D(s_{\alpha _1}, H)\cap D_\varGamma$. At step *i*, we insert arc $\alpha _i$ considering bisector $J(s_{\alpha _i}, {\tilde{H}}_{\alpha _i})$, where $H\supseteq {\tilde{H}}_{\alpha _i}\supseteq H_{\alpha _i}$, and $|{\tilde{H}}_{\alpha _i}|\le |H_{\alpha _i}|+2$. We use ${\tilde{H}}_{\alpha _i}$, possibly a superset of $H_{\alpha _i}$, in order to include at most one site in *H* for each neighbor of $\alpha _i$ in $\mathcal {P}_i$. This is done to correctly link two neighboring order-*k* arcs on $\mathcal {P}_i$ so that they are both incident to a common (new) Voronoi vertex. By Lemma 7.1, domain $D_i$ is a superset of the domain we would get if we instead considered bisector $J(s_{\alpha _i}, H)\supset \alpha _i$. Therefore, the relaxed construction works correctly. At the end, $D_h=f$.

We conclude that Theorem 6.13 applies, constructing  in expected time $O(|\partial f|)$.

Since the complexity of $\mathcal {V}_k(S)$ is $O(k(n-k))$, the $O(k^2(n-k) + n\log n)$ bound for iteratively constructing the diagram, starting at $\mathcal {V}(S)$, easily follows for $k\le n/2$. Although there are algorithms of better time complexity to construct $\mathcal {V}_k(S)$, such as the $O(k(n-k)\log ^2 n + n\log ^3 n)$ randomized incremental algorithm of Bohler et al. [5], the iterative construction is nice and simple, therefore, it can be preferable for small values of *k*.

## The Farthest Abstract Voronoi Diagram

In this section we show how to modify (in fact simplify) the algorithm for the deletion of one site to compute the *farthest* abstract Voronoi diagram, after the sequence of its faces at infinity is known.

The *farthest Voronoi region* of a site $p \in S$ is $\text{ FVR }(p,S) = \bigcap _{q \in S \setminus \{p\}} D(q,p)$ and the *farthest abstract Voronoi diagram* of *S* is $\textrm{FVD}(S) = {\mathbb {R}}^2\setminus \bigcup _{p \in S}\text{ FVR }(p, S)$. $\textrm{FVD}(S)$ is a tree of complexity *O*(*n*), however, regions may be disconnected and a farthest Voronoi region may consist of $\varTheta (n)$ disjoint faces [16]. Let $D^*(p,q)=D(q,p)$; then $\text{ FVR } (p,S) = \bigcap _{q \in S \setminus \{p\}}D^*(p,q)$.

Unless otherwise noted, we adopt the following convention: we reverse the labels of bisectors and use $D^*(\,{\cdot }\,,\,{\cdot }\,)$, in the place of $D(\,{\cdot }\,,\,{\cdot }\,)$, in most definitions and constructs of Sects. 3 and 4. Under this convention the definition of e.g., a *p*-monotone path remains the same but it uses $\partial \text{ FVR }(p,\,{\cdot }\,)$ in the place of $\partial {{\,\textrm{VR}\,}}(p,\,{\cdot }\,)$. The corresponding arrangement of *p*-related bisectors $\mathcal {J}_{p,S'}$, $S'\subseteq S$, is considered with the labels of bisectors and their dominance regions reversed from the original system $\mathcal {J}$.

Consider the enclosing curve $\varGamma$ as defined in Sect. 2, and let  be the sequence of arcs on $\varGamma$ derived by $\varGamma \cap \textrm{FVD}(S)$.  represents the sequence of the farthest Voronoi faces in $\textrm{FVD}(S)$ at infinity. The domain of computation is $D_\varGamma$. For an arc $\alpha$ of , let $s_\alpha$ denote the site in *S* for which $\alpha \subset \text{ FVR }(s_\alpha , S)$. With respect to site occurrences,  is a Davenport–Schinzel sequence of order 2.  can be computed in time $O(n\log n)$ in a divide and conquer fashion, similarly to computing the *hull* of a farthest segment Voronoi diagram, see e.g., [19].

We treat the arcs in  as sites and compute . Let  denote the face of $\textrm{FVD}(S)\cap D_\varGamma$ incident to , see Fig. 39.  is a tree whose leaves are the endpoints of the arcs in .

Consider , and let $S'\subseteq S$ be the set of sites that define the arcs in .

**Fig. 39:**
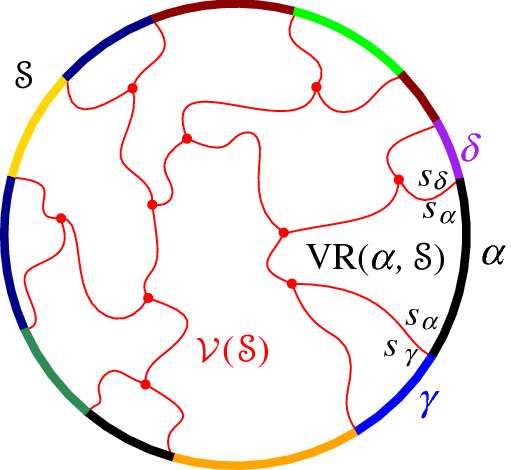
The farthest Voronoi diagram  and the Voronoi region . Bisector labels are shown in the farthest (reversed) sense

### Definition 8.1

A *boundary curve* $\mathcal {P}$ *for*  is a partitioning of $\varGamma$ into *arcs* by the bisector system $\mathcal {J}_{s,S'}$, such that any two consecutive arcs $\alpha ,\beta \in \mathcal {P}$ are incident to $J(s_\alpha ,s_\beta )\in \mathcal {J}_{s,S'}$, having *consistent labels*, and $\mathcal {P}$ contains an arc $\alpha \supseteq \alpha ^*$, for every core arc . We say that the labels of $\alpha ,\beta$ are consistent, if there is a neighborhood $\tilde{\alpha }\subseteq \alpha$ and $\tilde{\beta }\subseteq \beta$ incident to the common endpoint of $\alpha$ and $\beta$ such that $\tilde{\alpha }\in D^*(s_\alpha ,s_\beta )$ and $\tilde{\beta }\in D^*(s_\beta , s_\alpha )$.

There can be several different boundary curves for . The arcs in $\mathcal {P}$ that contain a core arc in  are called *original* and any remaining arcs are called *auxiliary*. The arcs in $\mathcal {P}$, although they are arcs on $\varGamma$, they are all boundary arcs and none is considered a $\varGamma$-arc in the sense of the previous sections. The endpoint $J(s_\alpha ,s_\beta )\cap \varGamma$ on $\mathcal {P}$ separating two consecutive arcs $\alpha ,\beta$ is denoted by $\nu (\alpha ,\beta )$.

The Voronoi-like diagram of a boundary curve $\mathcal {P}$ is defined analogously to Definition 3.3. Since $\mathcal {P}$ consists only of boundary arcs, $\mathcal {V}_l(\mathcal {P})$ is a tree whose leaves are the vertices of $\mathcal {P}$. The properties of a Voronoi-like diagram in Sect. 3 remain the same (under the conventions of this section).

Given $\mathcal {V}_l(\mathcal {P})$ for a boundary curve $\mathcal {P}$ of , we can insert a core arc  and obtain $\mathcal {V}_l(\mathcal {P}\oplus \beta ^*)$. The insertion is performed analogously to Sect. 4. The original arc $\beta \supseteq \beta ^*$, with endpoints *x*, *y* is defined as follows: let $\delta$ be the first arc on $\mathcal {P}$ counterclockwise (resp. clockwise) from $\beta ^*$ such that $J(s_\beta ,s_{\delta })\cap \delta \ne \emptyset$; let $x=\nu (\delta ,\beta )$ (resp. $y=\nu (\beta ,\delta )$). Let $\mathcal {P}_\beta =\mathcal {P}\oplus \beta$ be the boundary curve obtained from $\mathcal {P}$ by substituting with $\beta$ its overlapping piece from *x* to *y*. No original arc of $\mathcal {P}$ can be deleted by the insertion of $\beta$. Observation 4.1 remains the same, except from cases (d) and (e) which do not exist.

The *merge curve* $J(\beta )$, given $\mathcal {V}_l(\mathcal {P})$, is defined analogously to Definition 4.2; it is only simpler as it does not contain $\varGamma$-arcs. Theorem 4.3 remains valid, i.e., $J(\beta )$ is an $s_\beta$-monotone path in $\mathcal {J}_{s_\beta ,S'}$ connecting the endpoints of $\beta$. The proof structure is the same as for Theorem 4.3, however, Lemma 4.11 now requires a different proof, which we give in the sequel (see Lemma 8.3). Lemma 4.12 is not relevant; while Lemmas 4.13 and 4.14 are analogous.

In the following lemma we restore the labeling of bisectors to the original.

### Lemma 8.2

In an admissible bisector system $\mathcal {J}$, there cannot be two *p*-cycles, $p\in S$, with disjoint interior.

### Proof

By its definition, the nearest Voronoi region ${{\,\textrm{VR}\,}}(p,S)$ (resp. ${{\,\textrm{VR}\,}}(p,S)\cap D_\varGamma$) must be enclosed in the interior of any *p*-cycle of the admissible bisector system $\mathcal {J}$ (resp. $\mathcal {J}\cup \{\varGamma \}$). But ${{\,\textrm{VR}\,}}(p,S)$ (resp. ${{\,\textrm{VR}\,}}(p,S)\cap D_\varGamma$) is connected (by axiom (A1)), thus, there cannot be two different *p*-cycles with disjoint interior. $\square$

### Lemma 8.3

Consider the merge curve $J(\beta )$. Suppose $v_{i+1}$ is not a valid vertex because $v_{i+1}\in \alpha _{i}$, i.e., $e_i$ hits arc $\alpha _i$. Then vertex $v_{m-j}$ cannot be on $\mathcal {P}$.

### Proof

Suppose otherwise, i.e., vertex $$v_{m-j}$$ is on the boundary arc $$\alpha _{m-j}$$. Then $$J_x^i $$ and $$J_y^j$$ partition $$D_\varGamma $$ in three parts: a middle part incident to $$\beta $$, and two parts $$C_1$$ and $$C_2$$ at either side of $$J_x^i $$ and $$J_y^j$$ respectively, whose closures are disjoint, see Fig. 40. But the boundaries of $$C_1$$ and $$C_2$$ are $$s_\beta $$-cycles in the admissible bisector system $$\mathcal {J}\cup \{\varGamma \}$$ contradicting Lemma 8.2. Note that here we use the original labels of bisectors, including $$\varGamma =J(s_\beta ,s_\infty )$$. $$\square $$

**Fig. 40:**
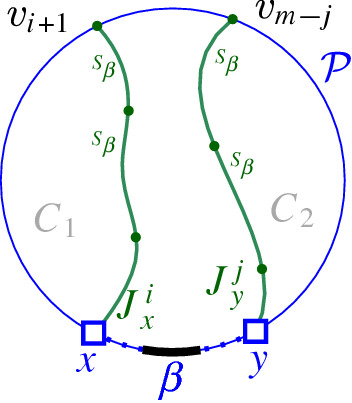
Illustration for Lemma 8.3. Nearest labels are shown

The diagram ${\mathcal {V}_l(\mathcal {P})\oplus \beta }$ is defined analogously and the proof that ${\mathcal {V}_l(\mathcal {P})\oplus \beta }$ is the Voronoi-like diagram $\mathcal {V}_l(\mathcal {P}_\beta )$ for $\mathcal {P}_\beta =\mathcal {P}\oplus \beta$, is analogous to the proof of Theorem 4.4.

The randomized algorithm for computing  is the same as in Sect. 6. The time analysis is also completely analogous. For completeness we point out that, here, the set $out _j$ consists of the auxiliary arcs in $\mathcal {B}_j$ that overlap with the auxiliary arcs of $\alpha _j$ in $\mathcal {B}_i$. The set $in _j$ are any remaining auxiliary arcs in $\mathcal {B}_j\setminus out _j$ that differ from the corresponding auxiliary arcs in $\mathcal {B}_i$. All observations of Sect. 6.1 remain intact under this updated notion of $in _j$ and $out _j$. Thus, the (expected) linear time complexity can be analogously established.

### Theorem 8.4

Given the sequence of its faces at infinity, i.e., given the sequence of arcs  implied by $\textrm{FVD}(S)\cap \varGamma$, the farthest abstract Voronoi diagram $\textrm{FVD}(S)$ can be computed in expected linear time .

## Concluding Remarks

In this paper we formalized the notion of an *abstract Voronoi-like diagram*, which is defined as a a tree (or forest) on the arrangement of the underlying bisector system related to a set of abstract sites *S*. We defined the Voronoi-like diagram of a *boundary curve*, which is implied by a subset of Voronoi edges bounding a Voronoi region ${{\,\textrm{VR}\,}}(s,S)$. We showed that the Voronoi-like diagram of a boundary curve is well defined, unique, and robust under an arc-insertion operation, which enables its use in incremental constructions. Using Voronoi-like diagrams as intermediate structures, we derived a very simple, randomized incremental algorithm to update an abstract Voronoi diagram, after deletion of one site, in expected linear time. The algorithm is applicable to any concrete diagram that falls under the umbrella of abstract Voronoi diagrams. In addition, the time complexity analysis offers a variant to backwards analysis, applicable to order-dependent structures.

The technique can be adapted to compute the order-$(k{+}1)$ subdivision within an order-*k* abstract Voronoi region, and the farthest abstract Voronoi diagram, after the order of its faces at infinity is known. The Voronoi-like structure provides the means to deal with the underlying disconnected Voronoi regions, which is the common complication of these, otherwise simple, Voronoi structures.

A deterministic linear-time construction of these diagrams remains an open problem. In future work we would like to consider Voronoi-like structures in the linear-time framework of Aggarwal et al. [1] aiming at a deterministic linear-time algorithm for the same problems.

## Footnotes

1 The presence of $\varGamma$ is conceptual and its exact position unknown; we never compute coordinates on $\varGamma$.
